# Reanalysis of *Wupus agilis* (Early Cretaceous) of Chongqing, China as a Large Avian Trace: Differentiating between Large Bird and Small Non-Avian Theropod Tracks

**DOI:** 10.1371/journal.pone.0124039

**Published:** 2015-05-20

**Authors:** Lida Xing, Lisa G. Buckley, Richard T. McCrea, Martin G. Lockley, Jianping Zhang, Laura Piñuela, Hendrik Klein, Fengping Wang

**Affiliations:** 1 School of the Earth Sciences and Resources, China University of Geosciences, Beijing, 100083, China; 2 Peace Region Palaeontology Research Centre, Tumbler Ridge, British Columbia, Canada; 3 Dinosaur Tracks Museum, University of Colorado Denver, Denver, Colorado, United States of America; 4 Museo del Jurásico de Asturias MUJA (Jurassic Museum of Asturias), Colunga, Spain; 5 Saurierwelt Paläontologisches Museum, Neumarkt, Germany; 6 Qijiang District Bureau of Land Resources, Chongqing, China; University of Pennsylvania, UNITED STATES

## Abstract

Trace fossils provide the only records of Early Cretaceous birds from many parts of the world. The identification of traces from large avian track-makers is made difficult given their overall similarity in size and tridactyly in comparison with traces of small non-avian theropods. Reanalysis of *Wupus agilis* from the Early Cretaceous (Aptian-Albian) Jiaguan Formation, one of a small but growing number of known avian-pterosaur track assemblages, of southeast China determines that these are the traces of a large avian track-maker, analogous to extant herons. *Wupus*, originally identified as the trace of a small non-avian theropod track-maker, is therefore similar in both footprint and trackway characteristics to the Early Cretaceous (Albian) large avian trace *Limiavipes curriei* from western Canada, and *Wupus* is reassigned to the ichnofamily Limiavipedidae. The reanalysis of *Wupus* reveals that it and *Limiavipes* are distinct from similar traces of small to medium-sized non-avian theropods (*Irenichnites*, *Columbosauripus*, *Magnoavipes*) based on their relatively large footprint length to pace length ratio and higher mean footprint splay, and that *Wupus* shares enough characters with *Limiavipes* to be reassigned to the ichnofamily Limiavipedidae. The ability to discern traces of large avians from those of small non-avian theropods provides more data on the diversity of Early Cretaceous birds. This analysis reveals that, despite the current lack of body fossils, large wading birds were globally distributed in both Laurasia and Gondwana during the Early Cretaceous.

## Introduction

**Fig 1:**
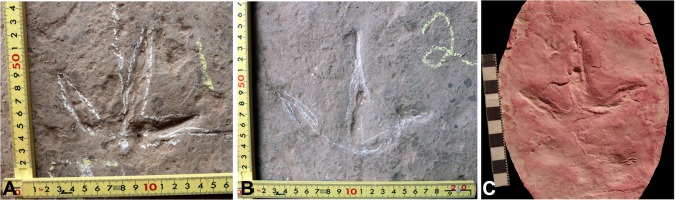
Footprints of *Wupus agilis* and *Limiavipes curriei*. *Wupus agilis* (A, B) and *Limiavipes curriei* (c). **A,** Print A6-1 (see S1 Table for footprint labels) of *W*. *agilis*. **B,** Print A6-2 of *W*. *agilis*. **C,** Replica of holotype RTMP 1998.089.0011 of *Limiavipes curriei* deposited at the PRPRC [3], [12]. Scale in centimeters.

**Fig 2:**
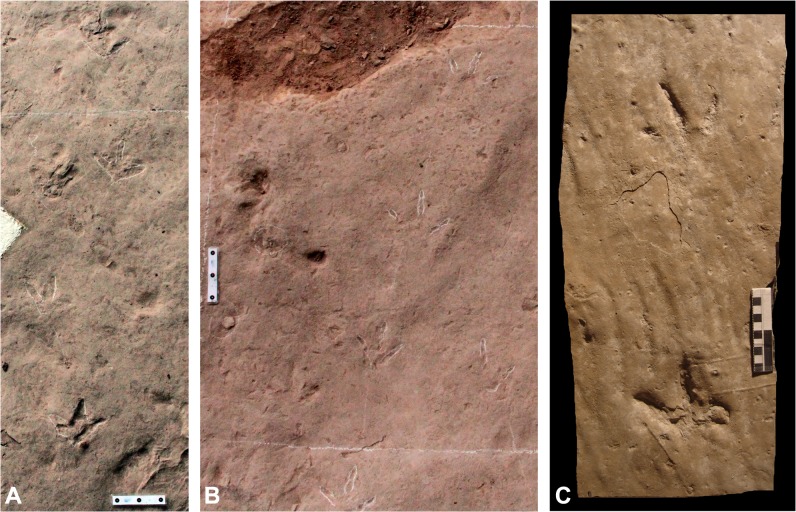
Trackway comparison of *Wupus agilis* and *Limiavipes curriei*. Two trackway segments (A-B) of *Wupus agilis* from the Lotus Stockade Tracksite. Note the short pace and stride relative to footprint length. **C,** PRPRC 2005.07.002, trackway of *Limiavipes curriei* (modified from McCrea et al. [3]). Scale = 10 cm.

In 2006, the Qijiang County Bureau of Land and Resources in Chongqing and the Southeast Sichuan Geological Team discovered dinosaur tracks within the Early to “middle” Cretaceous Jiaguan Formation that outcrops on Laoying Mountain, near the town of Sanjiao, Qijiang County. Xing et al. [1] assigned most of these tracks to Hadrosauriformes (*Caririchnium lotus*), and *Wupus agilis* (Figs 1A and 1B; 2A and 2B), a small tridactyl track type attributed to a small non-avian theropod track-maker [1]. Thereafter, Xing’s team found more sauropod and pterosaur (*Pteraichnus* isp.) ichnites [2]. Re-examination of *Wupus agilis* in 2012 by the authors indicates the prints are similar to those of *Limiavipes curriei* (Figs 1C; 2C). Herein we reassign *Wupus agilis*, originally ascribed to a non-avian theropod track-maker, to the ichnofamily Limiavipedidae [3] in recognition of the avian affinity of its track-maker. We also provide simple quantitative analyses that aid in differentiating between the traces of large avians and those of small non-avian theropod track-makers. For the sake of nomenclatural clarity, hereon “theropod” refers specifically to non-avian Theropoda outside of Aves, whereas “avian” refers to those Theropoda within Aves, following Gauthier [4]. This is necessary to clarify, as all Aves are Theropoda, but not all Theropoda are Aves.

### Institutional Abbreviations

QJGM, Exhibition Hall of Qijiang County Bureau of Land and Resources, Chongqing, China; PRPRC, Peace Region Palaeontology Research Centre, Tumbler Ridge, British Columbia, Canada; RTMP, Royal Tyrrell Museum of Palaeontology, Drumheller, Alberta, Canada.

## Materials and Methods

### Footprint and trackway data collection

**Fig 3:**
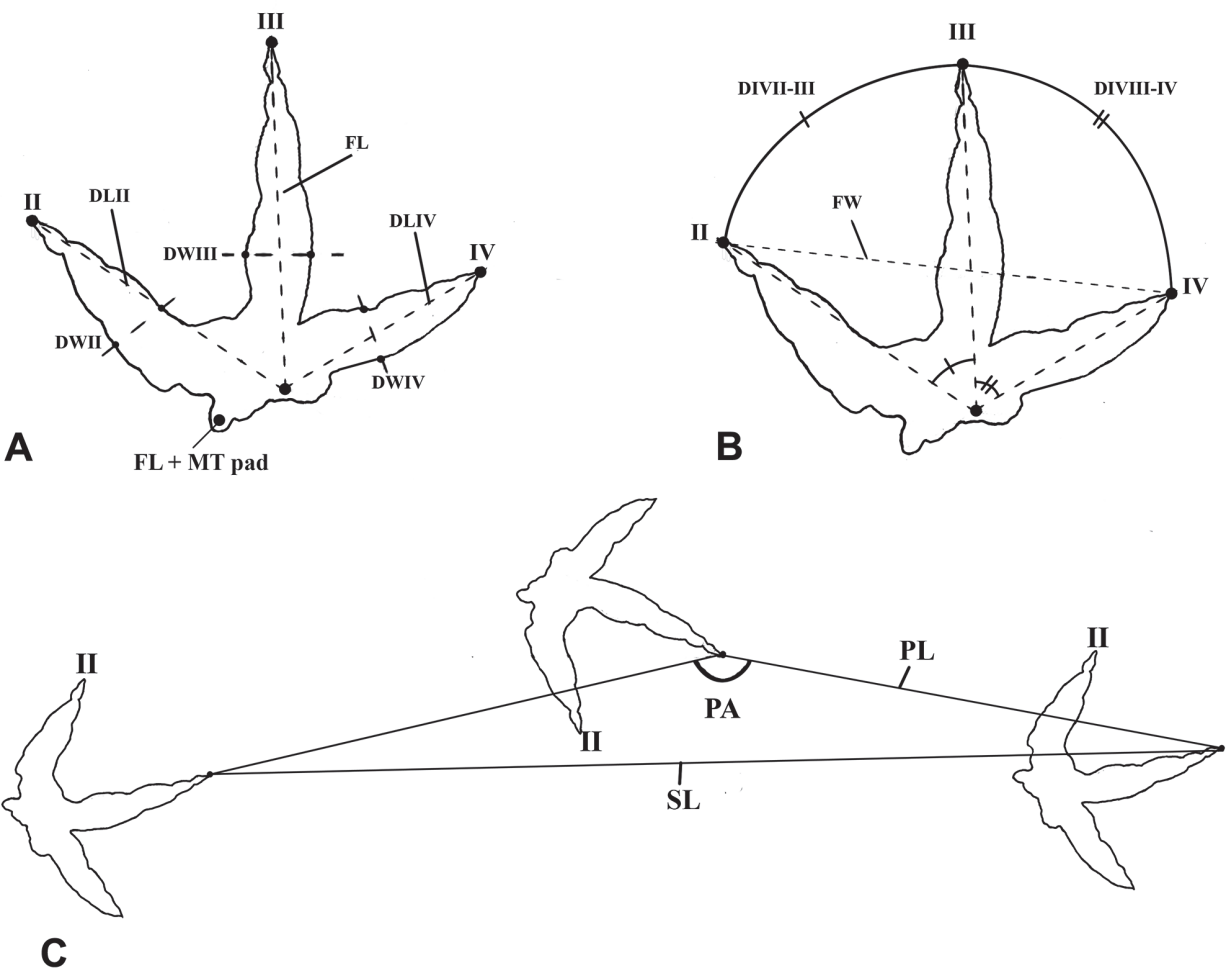
Data collection schematic for reanalysis of *Wupus agilis*. Diagrammatic representation of linear and angle measurements collected directly from individual prints (A,B) and trackways (C) of *Wupus agilis* (S1 Table and S2 Table). **A**, Footprint measurements: **II**, digit II; **III**, digit III; **IV**, digit IV; **FL**, footprint length; **DLII**, digit II length; **DLIV**, digit IV length; **DWII**; digit II width; **DWIII**, digit III width; **DWIV**, digit IV width. **B**, **DIVII–III**, digit divarication II–III; **DIVIII–IV**, digit divarication III–IV; **FW**, footprint width; **C**, Trackway measurements: **PL**, pace; **PA**, pace angulation; **SL**, stride. DIVTOT (not shown), total divarication, summed from measurements of DIVII–III and DIVIII–IV.

**Fig 4:**
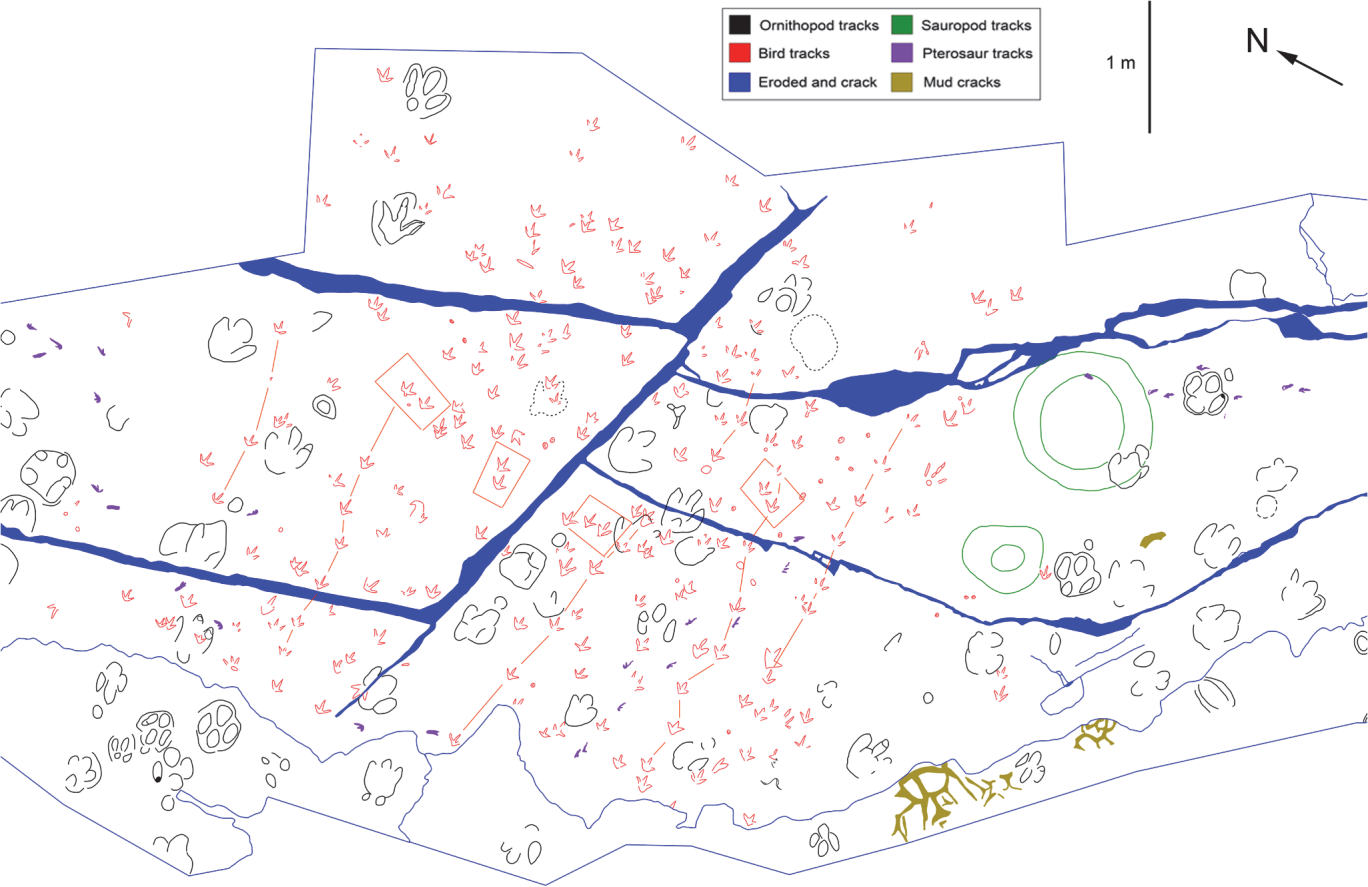
Trackway map of the Lotus Tracksite. Trackway map of the lower layer of the Qijiang locality, showing individual footprints and trackways of *Wupus agilis*, also showing pterosaur trackways of Xing et al. [2].

One hundred eighty three prints of *Wupus agilis* were re-examined at the Lotus Stockade and Tracksite in Chongqing, China in November 2012 (S1 and S2 Tables). Measurements were collected directly from the *in situ* prints. To avoid introducing assumptive errors in the data, prints that were not part of a discernible trackway were not identified as either left or right, and lateral digits in relation to digit III were measured as left digit (LD) and right digit (RD); however, where it was clear from the morphology of the track which digits were digit II (DII) and digit IV (DIV), those labels were used (Fig 3). A 1 meter x 1 meter grid was established along magnetic north-south and east-west lines on the track surface, and the tracks were traced on to a single sheet of acetate (Fig 4). Individual prints were numbered according to the grid square they occupied (e.g. the second print found in grid A5 is labeled A5-2; S1 and S2 Tables). No permits were required in order to conduct this research.

### Statistical analyses

Bivariate and multivariate analyses were performed on linear (FL, FW, DLII, DLIV, PL, SL,) and angular (DIVTOT, PA) data using PAleontological STatistics (PAST) version 3.0 [5]. Digit maximum width (DW) data were also collected (S1 Table), but were not used in the analyses as this metric was not often collected or reported in previous analyses and including this metric in the multivariate analyses would introduce a large amount of missing data. Other data that were collected (S1 Table) but were not used in the analyses were digit III length, and divarication angles between digits II–III and III–IV (DIVII–III; DIVIII–IV). This was done to compensate for the disparities in data collection and reporting for past studies of avian footprints. Digit III length is sometimes used as a proxy for footprint length, and in many instances divarication angles of digits II–III and digits III–IV are not reported. As the *Wupus agilis* and *Limiavipes curriei* samples are not reported with halluces, footprint length with hallux (FLwH) was also not used in the analyses. Data were log_10_-transformed and means were removed [6] prior to analysis to reduce the effects of absolute size on the results. Analyses performed were the t-test (bivariate), discriminant, and canonical variate. Discriminant analysis (DA) projects a multivariate data set down to one dimension in a way that maximises separation between *a priori* separated groups: in this case, the *a priori* groups are ichnotaxonomic groups of footprints attributed to avian or theropod track-makers. The *p*_same_ between two *a priori* groups was determined using Hotelling’s t^2^ test, the multivariate version of the t-test [5], [7] to determine significance at *p* ≥ 0.05.

## Geologic Setting

**Fig 5:**
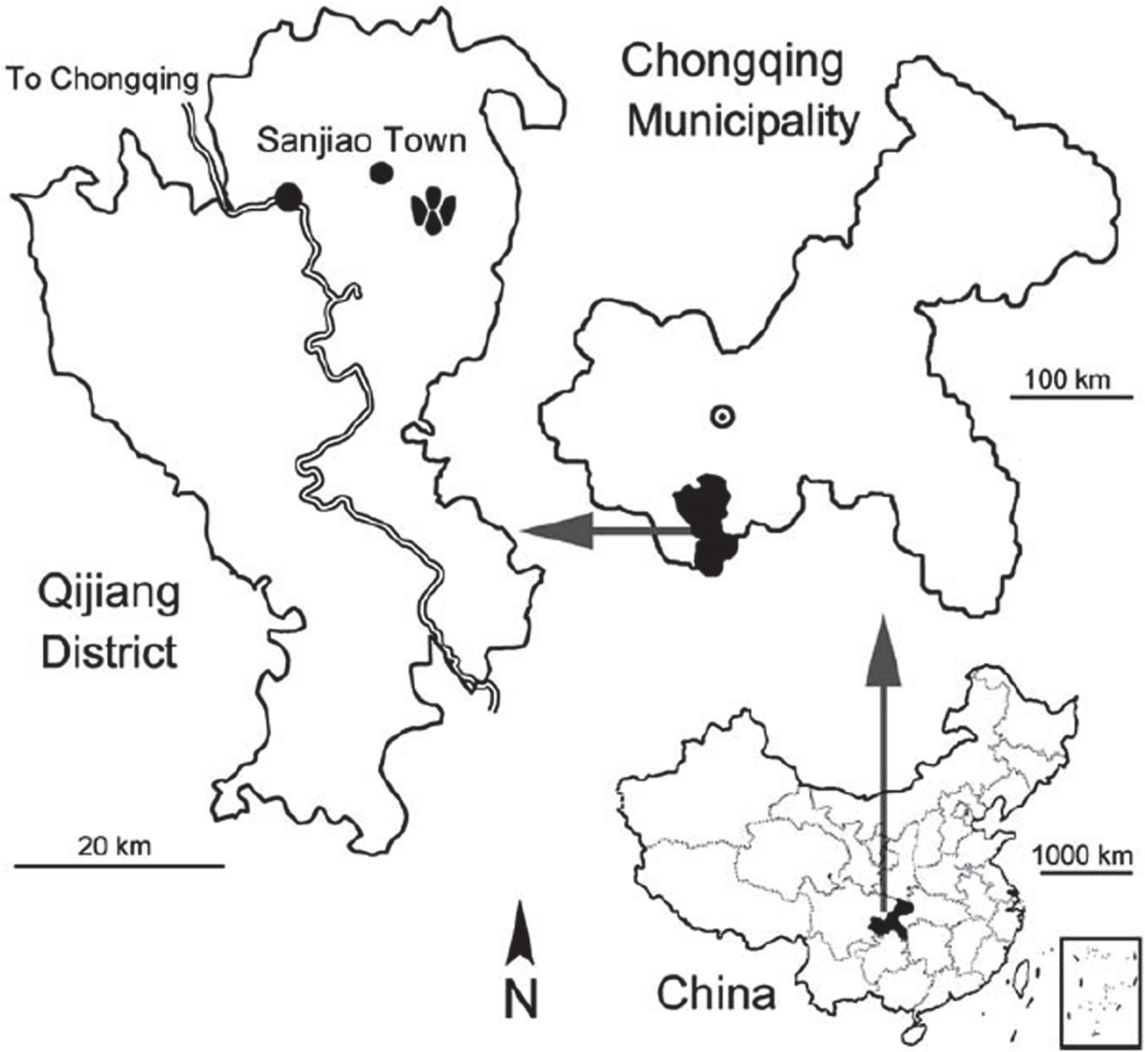
Geographic location of the *Wupus agilis* locality. Geographic map of the location (footprint icon) of the Qijiang locality (Lotus Tracksite) in Qijiang District, Chongqing Municipality, China. Modified from Xing et al. [2].

**Fig 6:**
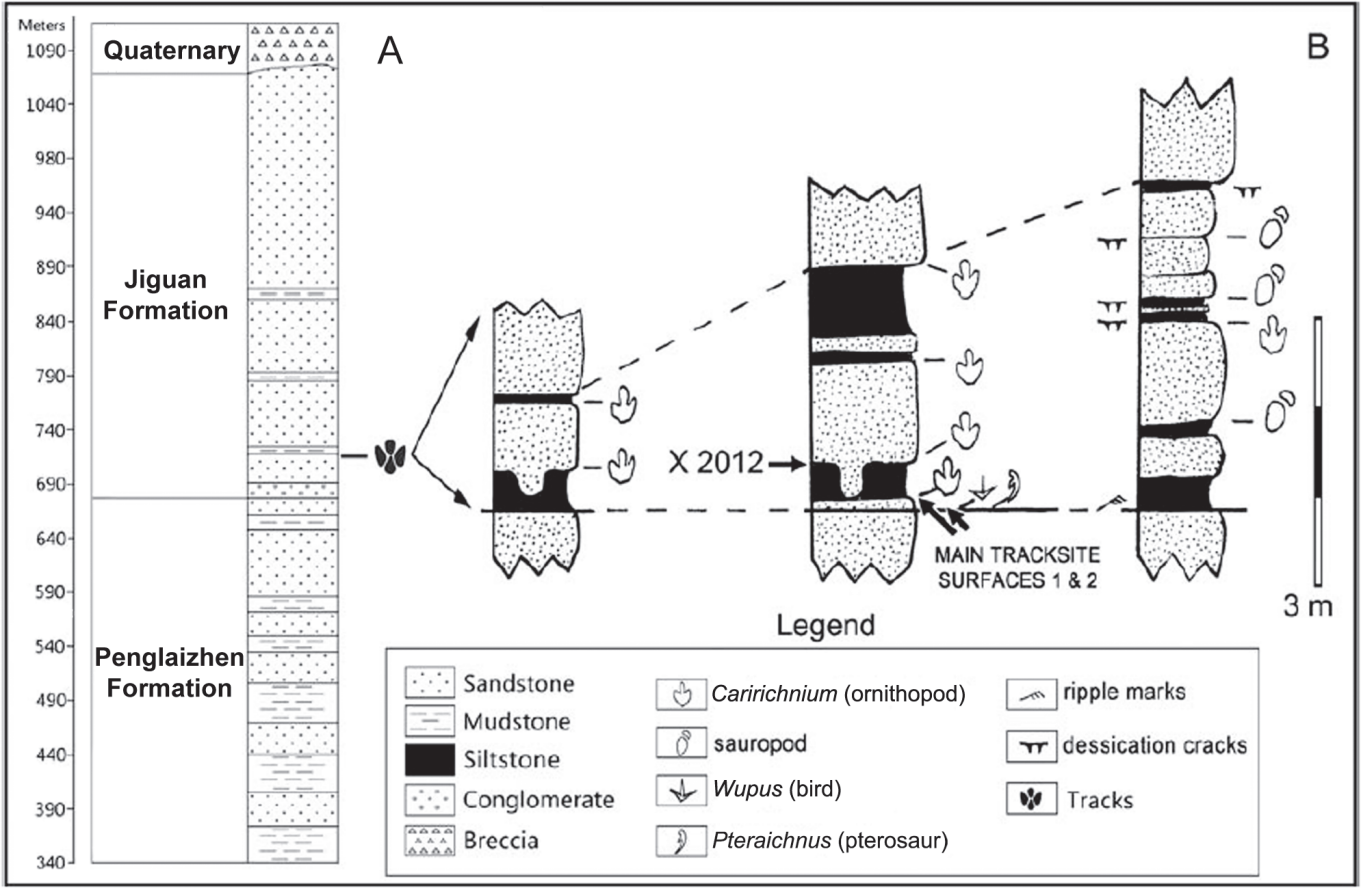
Stratigraphic section of the *Wupus agilis* locality. Stratigraphic section of the Jiguan Formation (left) with a detailed stratigraphic section of the Qijiang locality (right). Modified from Xing et al. [2].

The stratigraphic section within the Qijiang National Geologic Park (Fig 5) is described in detail in [2]. Specifically, the stratigraphic section of the Qijiang Tracksite includes the Upper Jurassic Pengliazhen Formation and the “middle” Cretaceous Jiaguan Formation [8], with 340 m of Pengliazhen Formation overlain above a parallel unconformity by 390 m of Jiaguan Formation. Qijiang Layer 1, the layer containing the *Wupus*-*Pteraichnus* ichnoassemblage of Xing et al. [2], occurs 30–40 m above the base of the Jiaguan Formation. The age of the Jiaguan Formation was calculated to be between 117 Ma and 85 Ma (Aptian–Santonian) [8] and between 140 and 85 Ma (Berriasian–Santonian) [9]. Recent pollen studies [10] support the latter age and it is adopted in this paper. The stratigraphy of the track-bearing layers of the Jiaguan Formation show an alternation of thin to thick bedded and massive sandstones with fluvial cross bedding, and blocky fine-grained siltstones and mudstones [2] (Fig 6). Many of the sandstones are lenticular and contain rip up clasts of the underlying siltstones and mudstones. Some of the sandstone surfaces display current ripples [2]. Deep desiccation cracks are common in siltstones [2].

## Results

### Ichnotaxonomic reassignment of *Wupus agilis* to an avian ichnofamily

*Wupus agilis* was originally attributed by Xing et al. [1] to a theropod track-maker based on the characters of coelurosaur prints of Thulborn [11]: foot length usually does not exceed 20 cm, maximum foot length is usually larger than maximum width, and the divarication angle between digits II and III is approximately the same as the that between digits III and IV. In the specific case of *Wupus*, the mean footprint length is 7.89 cm, and the mean footprint length to footprint width ratio (FL/FW) is 0.89, demonstrating that the footprint width exceeds footprint length. *Wupus* is morphologically similar to *Limiavipes curriei* ([3], emended from *Aquatilavipes curriei* [12]; Figs 1C and 2C), from the Grande Cache Member of the Gates Formation (Early Cretaceous: Albian). The morphologic similarity between *Wupus agilis* and *Limiavipes curriei* was noted [3], prompting a reanalysis of the attribution of *Wupus* as a non-avian theropod trace, and a reassignment of *Wupus* to the avian ichnofamily Limiavipedidae (see Systematic Ichnology.)

#### Differentiating between a large avian and a small non-avian theropod track-maker

Given the general morphological similarities between the tracks attributed to large birds and those of small theropods (bipedal, functionally tridactyl, tapering or sharp claws), criteria are required to distinguish the tracks of large avians from those of small theropods. Lockley et al. [13] list the criteria used to distinguish fossil avian tracks: 1) similarity to those of extant birds; 2) small size (although there are many extant birds with prints that are similar in size to those of small theropods); 3) slender digit impressions, with indistinct differentiation of digital pad impressions (although this is a factor of preservation and substrate consistency: trackways of extant birds can and do exhibit both prints with and without digital pads); 4) wide divarication angle (approximately 110°–120°) between digits II and IV (although there is a large amount of variation in the divarication of extant birds, and many prints display divarication below 110°); 5) a posteriorly directed hallux; 6) slender claws (Lockley et al. [14] use acuminate claws in their diagnosis of *Shandongornipes muxiai* as avian); 7) distal curvature of lateral (II and IV) claws away from the central axis of the foot; 8) track density; 9) associated fossils and feeding behavior; 10) sedimentological evidence regarding track-bearing deposits, such as trackway orientation to ripple crests, etc. In contrast, ichnites attributed to theropod track-makers possess 1), unequal lengths of digits II and IV, with digit IV being longer than digit II, 2) an average total divarication between lateral digits of 90° or less, 3) the theropod “notch”, or indent, in the proximal margin of the print in the region of the metatarsophanageal pad proximal to digit II, and 4) unguals of digits II and III curve medially, while the ungual of digit IV curve laterally [15].

As the dimensions of *Wupus agilis* and *Limiavipes curriei* overlap with those of small theropods (Tables 1–4), this requires that *Wupus agilis* be compared to traces of similarly-sized small- medium-, and large-sized theropods, as well as those traces of large Mesozoic, Cenozoic and extant avians. To determine whether the traces of both *Wupus agilis* and *Limiavipes curriei* share more affinities with large avians than with traces of small theropods, the linear and angular data of Limiavipedidae were compared to those of small- (*Irenichnites gracilis* [16]), medium- (*Columbosauripus ungulates* [16], *Magnoavipes lowei* [17], *Magnoavipes caneeri* [18], *Magnoavipes denaliensis* [19]) and large-sized (*Irenisauripus mcclearni* [16]), theropod trackways from the Cretaceous of North America, and to traces attributed to large avians from the Mesozoic (*Archaeornithopus* isp. [20], *Sarjeantopes* isp. [21]) and Cenozoic (*Gruipeda* isp. [22], [23], *Culcipeda* isp. [24], *Fuscinapeda* isp. [22], [23], *Leptoptilostipus* isp. [25], *Anatipeda* isp. [22], [23], [24], *Ardeipeda* isp. [22], [23], *Pavoformipes* isp. [26], *Ornothotarnocia* isp. [24], [27]) whose footprint lengths fall within the ranges of *Wupus agilis* and *Limiavipes curriei*. Also included in the analyses are data from the traces of the large, extant wading avians *Ardea herodias* (PRPRC NI2014.001, PRPRC NI2014.002) and juvenile *Branta canadensis* (PRPRC NI2014.004) from northeast British Columbia, Canada.

**Table 1:** Comparison of footprint lengths of the Limiavipedidae, theropod traces, and traces of Mesozoic, Cenozoic, and extant birds.

Track-maker	ichnotaxon	mean FL (mm)	minimum FL (mm)	maximum FL (mm)	Standard error, N
Limiavipedidae	*Wupus*	102	70	137	1, 160
	*Limiavipes*	78.9	63	101	1.2, 55
Theropod	*Irenichnites*	164.1	135	190	4.7, 11
	*Magnoavipes*	196.8	170	230	3.1, 25
	*Columbosauripus*	249.6	220	280	5.1, 13
	*Irenesauripus*	461.3	380	495	10.1, 13
Mesozoic bird	*Archaeornithipes*	120	75	166	26.3, 3
Cenozoic bird	*Leptoptilostipus*	94.0	80	115	2.8, 12
	*Culcipeda*	91.3	61	105	10.3, 4
	*Anatipeda*	65.5	58	73	2.7, 5
	*Gruipeda*	124	75	172	49, 2
	*Fuscinapeda*	98	96	100	2, 2
Extant bird	*Ardea herodias*	120	115	123	0.9, 8
	*Branta canadensis*	103	98	108	2, 4

Comparison of footprint lengths (mm) of *Wupus agilis* to *Limiavipes curriei* (Limiavipedidae), and to Cretaceous theropod traces *Irenichnites gracilis* (small), *Columbosauripus ungulates* and *Magnoavipes* (*M*. *lowei*, *M*. *caneeri*, *M*. *denaliensis*) (medium), *Irenesauripus mcclearni* (large), and traces of large Mesozoic, Cenozoic, and extant avians. If using size alone as a diagnostic criterion to distinguish small theropod traces from those of large avians, *Wupus agilis*, *Limiavipes curriei*, and *Irenichnites* isp. fall within a similar size class of track-maker as that of *Ardea* and *Gruipeda isp*.

**Table 2:** Comparison of footprint length to footprint width (FL/FW) ratios of the Limiavipedidae, theropod traces, and traces of Mesozoic, Cenozoic, and extant birds.

Track-maker	Ichnotaxon	mean FL/FW	min FL/FW	max FL/FW	Standard error, N
Limiavipedidae	*Wupus*	0.89	0.59	1.32	0.01, 144
	*Limiavipes*	0.76	0.59	0.94	0.01, 53
Theropod	*Irenichnites*	1.2	1.1	1.3	0.02, 11
	*Magnoavipes*	0.87	0.74	1.0	0.02, 24
	*Columbosauripus*	1.1	0.9	1.4	0.04, 12
	*Irenesauripus*	1.2	1.0	1.4	0.04, 12
Mesozoic bird	*Archaeornithipes*	1.0	0.99	1.0	0.01, 3
Cenozoic bird	*Leptoptilostipus*	0.95	0.89	1.02	0.01, 12
	*Culcipeda*	0.90	0.74	0.91	0.04, 4
	*Gruipeda*	1.01	0.96	1.07	0.06, 2
	*Fuscinapeda*	1.17	1.16	1.17	0, 2
	*Anatipeda*	0.92	0.84	1.04	0.04, 5
Extant bird	*Ardea herodias*	0.89	0.59	1.32	0.01, 144
	*Branta canadensis*	0.76	0.59	0.94	0.01, 53

Comparing footprint length to footprint width ratio (FL/FW) of *Wupus agilis* to the large avian trace *Limiavipes curriei* (Limiavipedidae) and to the Cretaceous theropod traces *Irenichnites gracilis* (small), *Columbosauripus ungulates* and *Magnoavipes* (*M*. *lowei*, *M*. *caneeri*, *M*. *denaliensis*) (medium), and *Irenesauripus mcclearni* (large), and traces of large Mesozoic, Cenozoic, and extant avians. There is considerable overlap in FL/FW values between theropod and avian traces, making FL/FW an unreliable metric when used alone to distinguish between large avian and small theropod footprints.

**Table 3:** Comparison of total divarication (also known as divarication between digits II-IV, DIVTOT) of Limiavipedidae, theropod traces, and traces of Mesozoic, Cenozoic, and extant birds.

Track-maker	ichnotaxon	mean DIVTOT (°)	minimum DIVTOT(°)	maximum DIVTOT(°)	Standard error, N
Limiavipedidae	*Wupus*	97.5	67	132	1.2, 147
	*Limiavipes*	125	107	150	2.0, 24
Theropod	*Irenichnites*	72	65	83	5.6, 3
	*Irenesauripus*	73.3	70	78	2.4, 4
	*Columbosauripus*	80.8	65	89	4.4, 5
	*Magnoavipes*	93.4	65	118	3.4, 23
Mesozoic bird	*Archaeornithipes*	113	70	150	23, 3
Cenozoic bird	*Leptoptilostipus*	?	?	?	?, 12
	*Culcipeda*	129	117	133	4, 4
	*Gruipeda*	96.5	72	121	24.5, 2
	*Fuscinapeda*	105	105	105	0, 2
	*Anatipeda*	92.4	84	98	3.4, 5
Extant bird	*Ardea herodias*	97.7	88	110	2.6, 9
	*Branta canadensis*	92.5	90	95	1.0, 4

Comparison of total divarication values (DIVTOT, also known as divarication between digits II–IV) values comparing *Wupus agilis* to *Limiavipes curriei* (Limiavipedidae), and Cretaceous small- (*Irenichnites* isp.) medium- (*Columbosauripus* isp., *Magnoavipes* isp.) and large-sized (*Irenesauripus* isp.) theropod ichnotaxa, and the traces of Mesozoic, Cenozoic and extant avians. While traces of large birds do overlap in some aspects of morphology with similarly-sized theropod traces, the mean total divarication of *Wupus agilis* is more similar to that of a large bird than that of a small theropod. While *Wupus agilis* is close in morphology to *Limiavipes curriei*, they are different enough in both size (Table 1) and total divarication to be considered distinct ichnotaxa.

**Table 4:** Comparison of footprint length to pace length (FL/PL) ratios of Limiavipedidae, theropod traces, and traces of Mesozoic, Cenozoic, and extant birds.

Track-maker	ichnotaxon	mean FL/PL	minimum FL/PL	maximum FL/PL	Number (N)
Limiavipedidae	*Wupus*	0.38	0.22	0.66	17
	*Limiavipes*	0.34	0.23	0.45	42
Theropod	*Irenichnites*	0.19	0.12	0.27	9
	*Irenesauripus*	0.31	0.27	0.40	12
	*Columbosauripus*	0.23	0.18	0.25	10
	*Magnoavipes*	0.18	0.14	0.21	17
Cenozoic bird	*Fuscinapeda*	0.44	0.43	0.45	2
Extant bird	*Ardea herodias*	0.47	0.30	0.78	5
	*Branta canadensis*	0.47	0.46	0.50	3

Comparing the footprint length (FL) to pace length (PL) ratios (FL/PL) of *Wupus agilis* to the large avian trace *Limiavipes curriei* (Limiavipedidae) and to small- (*Irenichnites* isp.) medium- (*Columbosauripus* isp., *Magnoavipes* isp.) and large-sized (*Irenesauripus* isp.) theropod ichnotaxa, and traces of large Cenozoic, and extant avians. *Wupus agilis* and *Limiavipes curriei* have a larger FL/PL than do small theropod traces of comparable size and those of medium-sized theropods. *Columbosauripus* isp. has the largest mean FL/PL of the analyzed theropod traces. The FL/PL of *Wupus agilis* and *Limiavipes curriei* have the most overlap with the FL/PL of avian traces. This indicates that, relative to the length of the track-maker’s foot, the track-makers of both *Wupus agilis* and *Limiavipes curriei* are either taking relatively shorter steps than similarly-sized theropods, or have relatively shorter legs than do similarly-sized theropods.

#### Total divarication/footprint splay and determination of theropod versus bird tracks

High divarication has been used to differentiate between traces of large avians and those of small theropods [17], [18], [19]. A total divarication of less than 90° is reported to be a feature in theropod prints [15], whereas a total divarication of greater than 100° has been used as an avian trace character [13]. In extant shorebirds, for example, total divarication (the divarication between digit II and digit IV) within a single trackway can range from 75.5°–116.5°, and have an average total divarication of 96.1° (PRPRC NI2011.003, *Tringa solitaria*, Solitary Sandpiper). In a large wading bird, *Ardea herodias*, comparable in size to Limiavipedidae, total divarication ranges from 88°–110° with an average total divarication of 97.7° (Table 3). Conversely, the small-, medium- and large-sized theropod traces range in total divarication from 65°–120°, with an average total divarication of 72° for tracks attributed to small theropods (*Irenichnites* isp.), 81° and 93° for tracks attributed to medium-sized theropods (*Columbosauripus* isp. and *Magnoavipes* isp., respectively), and 73° for tracks attributed to large-sized theropods (*Irenesauripus* isp.) (Table 3). Relying on total divarication alone would result in the tracks of large avians, such as traces of Cenozoic *Gruipeda* isp. and *Anatipeda* isp. from the Cenozoic, and traces of extant *Ardea herodias* and *Branta canadensis*, being classified as those of non-avian theropods if these traces were to be discovered in Mesozoic sediments, as these traces have an average total divarication of less than 100°. In general, total divarication of greater than 100° is a characteristic that can be used to differentiate between small theropod and large bird traces from the Cretaceous only when those divarications are preserved, and this is demonstratively unrealistic based on the ranges of total divarication of the traces of both Cenozoic and extant avians. Average total divarication alone is a potentially misleading feature due to the large degree of overlap between the tracks of large avian and small theropod track-makers.

#### Footprint Length to Pace Length Ratio (FL/PL)

The pace in avian trackways is, on observation, relatively shorter than that seen in similarly-sized theropod traces. In Lockley et al.’s [18] interpretation of *Magnoavipes* isp. as having been made by a theropod (rather than avian) track-maker, they note that the relatively long pace and low footprint rotation of *Magnoavipes* isp., despite the high total divarication (which can also be observed in the traces of ornithopod affinity), are more characteristic of theropod track-makers. To determine quantitatively if the pace in a bird trackway is relatively shorter than that of a theropod, the footprint length/pace length ratio (FL/PL) was calculated for those ichnotaxa for which the data were available (Table 4). The footprint length to pace length ratios (FL/PL) of *Wupus agilis* (0.38) and *Limiavipes curriei* (0.34) are on average larger when compared to the same ratios from small-sized (*Irenichnites* isp., 0.19), medium-sized (*Columbosauripus* isp., 0.23; *Magnoavipes* isp., 0.18), and large-sized (*Irenesauripus* isp., 0.31) theropod trackways from the Cretaceous of North America, although there is overlap in the range between the smallest and the largest ratio values (Table 4). The traces of *Wupus agilis* and *Limiavipes curriei* are closer in FL/PL to those of Cenozoic and extant avians: traces of *Fuscinapeda* isp. display the largest FL/PL ratio (0.44), while the FL/PL ratio of *Ardea herodias* is 0.30 and *Branta canadensis* is 0.47.

This metric indicates that, relative to the length of the track-maker’s foot, the track-makers of Limiavipedidae and large wading birds are either taking relatively shorter steps than theropods with similarly-sized pedes (due to either behavioral or biomechanical reasons), or have relatively shorter legs than do similarly-sized theropods. Both traces of *Limiavipes curriei* and *Wupus agilis* are preserved on fine-grained sandstone without any of the extramorphologic features (e.g. digit collapse, such as observed in *Magnoavipes* isp. [28], slide marks, etc.) or trackway features (e.g. high inward footprint rotation) that would indicate atypical or hampered movement, indicating that shortened steps are not an artifact of the original substrate composition.

Interpretation of the morphology and bivariate data of the traces of *Wupus agilis* and *Limiavipes curriei* (Figs 1 and 2) indicates that the Limiavipedidae share more characteristics with Cenozoic and extant avian track-makers than they do small- and medium-sized theropod track-makers, supporting the attribution of the Limiavipedidae to an avian track-maker.

#### Multivariate statistical analyses

**Fig 7:**
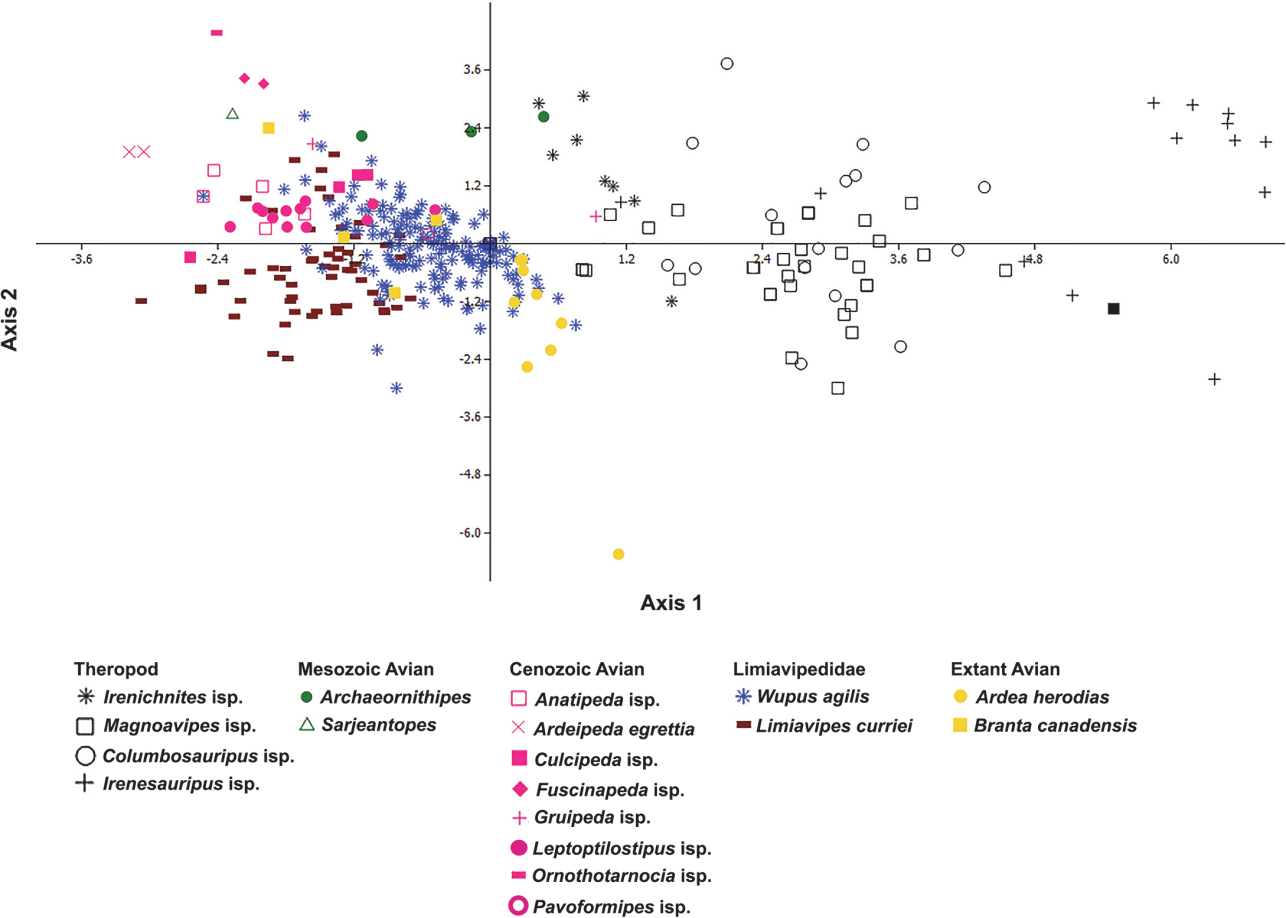
Discriminant analysis morphospace plot comparing Limiavipedidae (*Wupus* and *Limiavipes*) to prints of small- and medium-sized theropods and large wading birds. Discriminant analysis scatterplot comparing log_10_-transformed and mean removed linear data (footprint length, FL; footprint width, FW; digit II length, DLII; digit IV length, DLIV; pace length, PL; stride length, SL) and mean removed angular data (total divarication, DIVTOT; pace angulation, PA) of Limiavipedidae (*Wupus agilis*, dark blue; *Limiavipes curriei*, dark brown) to ichnotaxa of Cretaceous theropods (black), Mesozoic avians (green), Cenozoic avians (pink), and traces of extant avians (orange). The scatterplot shows that Limiavipedidae, as well as the Cenozoic avian ichnotaxa and ichnites from extant avians, do not share morphospace with Cretaceous theropod tracks. Axis 1 is interpreted as the size–total divarication axis; as size and pace angulation increase (as the size of the trackmaker increases and as the trackway narrows), total divarication decreases. This is consistent with the observations of theropods having a smaller total divarication, as well as a larger size and narrower trackway. Theropods group positively along Axis 1, while birds, with their smaller size and higher total divarication, and more “toed-in” footprints, group negatively along Axis 1. Axis 2 is interpreted as the relationship between FW and the lengths of the lateral digits to FL, PL, and SL; footprints with longer lateral digits (DII, DIV) are relatively shorter in length, and are found in trackways with shorter PL and SL. Avian prints are interpreted to have subequal lateral digits and a higher L/W ratio, and the discriminant analysis correlates with the interpretation that avian prints belong to trackways with a relatively shorter pace length (Tables 4–5).

Discriminant analysis on Limiavipedidae (*Wupus agilis*, *Limiavipes curriei*), small- (*Irenichnites* isp.), medium- (*Columbosauripus* isp., *Magnoavipes* isp.), and large-sized (*Irenesauripus* isp.) theropod ichnotaxa, Mesozoic (*Archaeornithipes* isp., *Sarjeantopes* isp.) and Cenozoic (*Leptoptilostipus* isp., *Culcipeda* isp., *Gruipeda* isp., *Fuscinapeda* isp., *Anatipeda* isp.) avian ichnotaxa, and data collected from the tracks of extant large avians (*Ardea herodias*, juvenile *Branta canadensis*) indicate that Limiavipedidae share a morphospace with the tracks of both Cenozoic and extant avians, but does not share morphospace with the theropod ichnites (Fig 7, Tables 5 and 6), forming distinct “theropod” and “avian” morphospace groups (*p*_same_ = 1.35 × 10^–84^, 97.6% correctly identified). None of the footprints of Limiavipedidae were mistakenly classified as belonging to the theropod group, whereas the analysis misclassified 48 tracks of Limiavipedidae as belonging to several Cenozoic avian ichnotaxa (seven *Leptoptilostipus* isp., 11 *Gruipeda* isp., 11 Anseriformes, ten *Culcipeda* isp., 12 *Ardea herodias*, with four prints identified as “bird”, Table 6). Also, seven theropod prints (one of *Irenesauripus* isp., six of *Magnoavipes* isp.) were misclassified as belonging to the Limiavipedidae, and were tracks that contain a large amount of missing data save for footprint length, and they could not be accurately placed by the analysis.

**Table 5:** Discriminant analysis loadings of variables for log10-transformed and mean removed data comparing Limiavipedidae to tracks of Cretaceous theropods and tracks of similarly-sized Mesozoic, Cenozoic, and extant avians.

Variable	Axis 1	Axis 2	Axis 3	Axis 4	Axis 5	Axis 6	Axis 7	Axis 8	Axis 9	Axis 10
FL	0.09183	0.023	-0.00691	0.0092	-0.02766	-0.02629	0.02272	-0.01653	-0.0168	2.07 × ^-238^
FW	0.07650	-0.01158	-0.02525	-0.00322	-0.00378	-0.00911	0.00965	0.02983	0.01507	-3.09 × ^-238^
L/W	0.02617	0.07674	0.02030	0.00829	-0.04501	0.01082	0.05779	-0.05761	0.03323	8.98 × ^-237^
DLII	0.03428	-0.04661	-0.05318	0.03537	-0.02105	-0.00197	0.05343	-0.02024	-0.00189	-1.81 × ^-237^
DLIV	0.03449	-0.05094	-0.01837	0.05394	-0.01649	-0.00643	0.05654	-0.00958	0.00558	-1.63 × ^-237^
DIVTOT	0.08207	0.05128	-0.02951	0.07749	0.0071	0.05795	-0.06175	-0.04006	0.01348	6.33 × ^-237^
PL	0.06178	0.01347	0.00442	-0.00742	0.09437	0.01807	-0.00463	-0.08623	0.01187	1.38 × ^-236^
SL	-2.0329	2.3819	-3.6663	1.1274	7.3197	-8.4611	-0.87416	6.8702	0.61591	7.97 × ^-235^
PA	0.68402	-0.2079	-0.16886	0.21129	-0.32365	-2.2594	-2.4202	-2.1587	2.6084	2.95 × ^-235^

Variable loadings for discriminant analysis comparing log_10_-transformed and mean removed linear data (footprint length, FL; footprint width, FW; digit II length, DLII; digit IV length, DLIV; pace length, PL; stride length, SL) and mean removed angular data (total divarication, DIVTOT; pace angulation, PA) of Limiavipedidae (*Wupus agilis*, *Limiavipes curriei*) to small- (*Irenichnites* isp.) medium- (*Columbosauripus* isp., *Magnoavipes* isp.) and large-sized (*Irenesauripus* isp.) theropod ichnotaxa, and Mesozoic (*Archaeornithipes* isp., *Sarjeantopus* isp.) and Cenozoic (*Leptoptilostipus* isp., *Culcipeda* isp., *Gruipeda* isp., *Fuscinapeda* isp., *Pavoformipes* isp.) avian ichnotaxa, and data collected from the tracks of extant large avians (*Ardea herodias*, juvenile *Branta canadensis*). Fig 7 shows the axes (axes 1 and 2) along which maximal separation of the grouped data occur. Axis 1 is interpreted as the effect of size on the variation present in the dataset; however, as size and pace angulation increase (as the size of the trackmaker increases and as the trackway narrows), total divarication decreases. This is consistent with the observations of theropods having a smaller total divarication, as well as a larger size and narrower trackway. Theropods group positively along Axis 1, while birds, with their smaller size and higher total divarication, and more “toed-in” footprints, group negatively along Axis 1. Axis 2 is interpreted as the relationship between FW and the lengths of the lateral digits to FL, PL, and SL; footprints with longer lateral digits (DII, DIV) are shorter in length, and are found in trackways with shorter PL and SL. Limiavipedidae group with Cretaceous avian traces and Cenozoic avian traces (Fig 7). Avian prints are interpreted to have subequal lateral digits and a higher L/W ratio, and the discriminant analysis correlates with the interpretation that avian prints belong to trackways with a relatively shorter pace length (Table 4).

**Table 6:** Confusion matrix from discriminant analysis of footprints of Limiavipedidae, theropods, and prints from large Mesozoic, Cenozoic, and extant birds.

		Predicted Group											
	*A priori* groups	Theropod	Limiavipedidae	*Archaeornithipes*	*Leptoptilostipus*	*Gruipeda*	Anseriform	*Culcipeda*	*Ardeipeda*	*Fuscinapeda*	*Ardea herodias*	Bird	Total
**Given Group**	**Theropod**	**61**	**7**	**1**	**0**	**8**	**0**	**0**	**0**	**0**	**3**	**0**	**80**
	**Limiavipedidae**	**0**	**188**	**1**	**7**	**11**	**11**	**10**	**0**	**0**	**12**	**3**	**243**
	***Archaeornithipes***	**0**	**0**	**1**	**0**	**0**	**1**	**0**	**0**	**0**	**0**	**1**	**3**
	***Leptoptilostipus***	**0**	**2**	**0**	**10**	**0**	**0**	**0**	**0**	**0**	**0**	**0**	**12**
	***Gruipeda***	**0**	**1**	**0**	**0**	**0**	**1**	**0**	**0**	**0**	**0**	**0**	**2**
	**Anseriform**	**0**	**0**	**0**	**0**	**0**	**7**	**0**	**1**	**0**	**1**	**0**	**9**
	***Culcipeda***	**0**	**0**	**0**	**0**	**0**	**0**	**4**	**0**	**0**	**0**	**0**	**4**
	***Ardeipeda***	**0**	**0**	**0**	**0**	**0**	**0**	**0**	**2**	**0**	**0**	**0**	**2**
	***Fuscinapeda***	**0**	**0**	**0**	**0**	**0**	**0**	**0**	**0**	**2**	**0**	**0**	**2**
	***Ardea herodias***	**0**	**2**	**0**	**0**	**0**	**0**	**0**	**0**	**0**	**7**	**0**	**9**
	**Bird**	**0**	**1**	**0**	**0**	**0**	**0**	**1**	**0**	**0**	**0**	**1**	**3**
	**Total**	**61**	**201**	**3**	**17**	**19**	**20**	**15**	**3**	**2**	**23**	**5**	**369**

Confusion matrix of discriminant analysis comparing log_10_-transformed and mean removed linear data (footprint length; footprint width;; digit II length; digit IV length; pace length, PL; stride length, SL) and mean removed angular data (total divarication; pace angulation) of Limiavipedidae (*Wupus agilis*, *Limiavipes curriei*) to small- (*Irenichnites* isp.), medium- (*Columbosauripus* isp., *Magnoavipes* isp.), and larg-sized (*Irenesauripus* isp.) theropod ichnotaxa, Mesozoic (*Archaeornithipes* isp., *Sarjeantopes* isp.) and Cenozoic (*Leptoptilostipus* isp., *Culcipeda* isp., *Gruipeda* isp., *Fuscinapeda* isp., *Pavoformipes* isp., *Ornothotarnocia* isp.) avian ichnotaxa, and data collected from the tracks of extant large avians (*Ardea herodias*, juvenile *Branta canadensis*). No individual footprints of Limiavipedidae were identified as theropod ichnites in the predicted groupings, while 48 prints of Limiavipedidae were misidentified as the avian ichnotaxa *Leptoptilostipus* isp., *Gruipeda* isp., *Culcipeda* isp., and “bird” (*Sarjeantopes* isp., *Pavoformipes* isp., *Ornothotarnocia* isp.), and the tracks of extant *Ardea herodias* and those of both extant and fossil Anseriformes.

Multivariate analysis of variance (MANOVA) was conducted on Limiavipedidae with the small-, medium-, and large-sized theropod ichnotaxa, the Cenozoic avian ichnotaxa, and prints from extant large avians with footprints similar in size to traces of Limiavipedidae. The ichnites with which Limiavipedidae were mistaken in the confusion matrix indicate that Limiavipedidae is significantly different from the theropod ichnites (*p*_same_ = 4.47 x 10^–27^) and is also significantly different (although to a lesser degree) from *Leptoptilostipus* isp. (*p*_same_ = 9.03 x 10^–16^), *Culcipeda* isp. (*p*_same_ = 6.61 x 10^–05^), Anseriformes (*p*_same_ = 7.39 x 10^–04^), *Fuscinapeda* isp. (*p*_same_ = 0.04), and extant *Ardea* (heron; *p*_same_ = 5.58 x 10^–04^). Limiavipedidae were not significantly different from the ichnites *Ardeipeda* isp. (*p*_same_ = 0.36) or *Gruipeda* isp. (*p*_same_ = 0.36). Multivariate statistical analyses comparing *Wupus* and *Limiavipes* to small- and medium-sized theropod ichnites, and to large avian ichnites from the Mesozoic, Cenozoic, and from extant avians reveals, 1) the similarity between *Wupus* with *Limiavipes*, providing additional support for the assignment of *Wupus* to Limiavipedidae, and 2) that the traces assigned to Limiavipedidae are more similar to those traces of large avians than they are to traces of small- and medium-sized theropods, and supports the attribution of Limiavipedidae to a large avian track-maker.

### Systematic Ichnology

Class Aves

Ichnofamily Limiavipedidae, McCrea et al. [3]

Diagnosis (from McCrea et al. [3]): Trackway of a large, long-legged avian track-maker. Functionally tridactyl pes track with no obvious webbing. No hallux impression. Digits with sharp, tapering claws. Digital pad impressions show phalangeal formula of? -2-3-4-0, with digit I impression likely not preserved. Mean of the total digit divarication over 100°. Pes width greater than length. Pace and stride short compared to similar-sized theropod ichnotaxa, but long compared to other Mesozoic avian ichnotaxa. Average pace greater than 20 cm, average stride greater than 38 cm. Strong rotation of the footprints toward the midline of the trackway (Figs 1 and 2).

Type Ichnogenus: *Limiavipes* isp., McCrea et al. [3].

Ichnogenus *Wupus*, Xing et al. [1]

Emended diagnosis: A large tridactyl avian footprint with no discernible hallux (Fig 1A and 1B); digital pads distinct with phalangeal formula of? -2-3-4-0; well-preserved prints display a sub-rounded metatarsal impression that is closer to digit II than digit IV; webbing indistinct; footprint length to footprint width ratio 0.89; divarication angle between digits II and III is approximately 50°, with a mean total divarication of under 100°, although total divarication can range 67°–132°; pace angulation is 180°; footprint length to pace length ratio 1:3.62 (Figs 1A and 1B; 2A and 2B).

Locality and horizon: Lotus Tracksite, Qijiang, Chongqing, Lower Cretaceous (Barremian—Albian), Jiaguan Formation, China.

Ichnospecies *Wupus agilis*, Xing et al. [1] (Figs 1A and 1B; 2A and 2B; S1 Table and S2 Table)

Emended diagnosis: as for ichnogenus.

Discussion: We no longer feel the original assignment by Xing et al. [1] of *Wupus agilis* as the trace of a small theropod is accurate given detailed comparisons of traces of *Wupus agilis* to those ichnotaxa of large birds and small theropods. *Wupus agilis* closely conforms to the diagnosis for Limiavipedidae [3]. Where well-preserved, digits II–IV terminate distally in short, acuminate claws (Fig 1A and 1B), a feature that is identified as avian by Lockley et al. [13].

Footprints of *Wupus agilis* often display a prominent ridge occupying the length of the long-axis of one or more digits. We regard these as indicators of substrate consistency, caused by suction or adhesion of the surficial mud-drape to the underside of the track-makers’ feet during the take-off phase of the pedes. The sediment is pulled up from the surface during the withdrawal of the track-maker’s feet and forms this extramorphological ridge within the digit impressions. This feature should not be regarded as having ichnotaxonomic significance, and is simply a consequence of walking on a damp, fine-grained surface. This feature is present in many tracks of *Limiavipes curriei* from western North America.

#### Retention of *Wupus* and *Limiavipes* as separate ichnotaxa

Comparison of *Wupus agilis* with *Limiavipes curriei* indicates that while there are many similarities, there are enough morphologic differences to retain *Wupus* isp. and *Limiavipes* isp. as distinct ichnotaxa. *Limiavipes curriei* has an average footprint length (FL) of 7.9 cm (6.3 cm—10.1 cm), while *Wupus agilis* is larger, with an average FL of 10.3 cm (7.0 cm—17.0 cm). In well-preserved prints of *Wupus agilis* the proximal margin of the pes is asymmetrically bi-lobed, displaying a short, postero-medially protuberance which may correlate with the metatarsal pad for an unimpressed hallux; however, no halluces were identified in 187 documented prints. The prints of *Wupus agilis* possess an average FL/footprint width (FW) of 0.9 (0.6–1.3), while those of *Limiavipes curriei* display a larger splay and possess a FL/FW of 0.75 (0.6–0.9) (Tables 1–2). The average total divarication (II-IV) of *Wupus agilis* is 96.9° (67°–132°), which is smaller than the average total divarication of *Limiavipes curriei* (123°). In four trackways, the average P and S lengths of are 38.7 cm (23 cm—63 cm) and 75.9 cm (48.5 cm—113.5 cm), respectively, and a FL to PL ratio of 0.3 (0.2–0.6) (Table 3; Fig 2). This is similar to *Limiavipes curriei*, where average pace and stride lengths are 23.9 cm (18 cm—31.5 cm) and 46.5 cm (36.5 cm—60.0 cm), and a FL/PL of 0.3 (0.2–0.4). *Wupus agilis* represents a larger track-maker in both footprint length and leg length with more narrowly-splayed pedes than that of *Limiavipes curriei* (Tables 3 and 4).

## Discussion

Ichnomorphology, multivariate statistical analyses, and interpretations of total divarication, footprint splay, FL/PL support that *Wupus agilis* is the most similar to *Limiavipes curriei* and can confidently be assigned to the ichnofamily Limiavipedidae. Multivariate statistical analyses of *Wupus agilis* and *Limiavipes curriei* with tracks of Cretaceous small- (*Irenichnites* isp.), medium- (*Magnoavipes* isp., *Columbosauripus* isp.), and large-sized (*Irenesauripus* isp.) theropods, and with large avian traces from the Cenozoic and those of extant birds, demonstrate that Limiavipedidae is distinct from similarly-sized small- and medium-sized traces attributable to theropods. Limiavipedidae occupies a similar morphospace to traces of the Cenozoic avian ichnotaxa *Leptoptilostipus* isp., *Culcipeda*, isp., *Gruipeda* isp., and *Fuscinapeda* isp. Ichnomorphology, multivariate statistical analyses, and interpretations of total divarication, footprint splay, FL/PL support the attribution of *Wupus agilis* and *Limiavipes curriei* to large, wading avian track-makers.

### Distinguishing between the tracks of small theropods versus large birds in the Cretaceous

Similarities in pes morphology between Cretaceous small- and medium-sized theropods and large wading birds can lead to multiple interpretations with respect to the potential track-maker. While it would be convenient if there were a single track or trackway feature exclusive to either small theropods or to large birds that is consistently preserved, nature is rarely so unambiguously dichotomous. Interpreting footprints requires addressing a high degree of natural variation in metrics inherent in the preserved movements of living animals. There are a number of different metrics that must be considered when attempting to make a distinction between tracks of avians and those of small theropod. There are many bird-like features in theropod dinosaur footprints, and many of the issues in distinguishing the tracks of bipedal tridactyl dinosaurs from those of avians may be due to similar functional constraints in locomotion [15] (although see Gatesy [29] and Farlow *et al*. [30] for differences between avian and theropod locomotor constraints.)

In short, there is no one feature that can be used to distinguish the print of a small theropod from that of a large wading bird 100% of the time; this particularly holds true when attempting to identify an isolated footprint or even a single trackway, as there can be a large amount of variability in digit splay and preservation of accurate digit lengths. Rather, a combination of features (size, divarication, digit splay, and footprint length to pace length ratio) should be used to distinguish between traces of small theropods and those of large wading avians.

#### Size alone does not a small theropod trace make

Comparisons of data (Tables 1–4) demonstrate that there is considerable overlap in the documented sizes of the avian prints of *Wupus agilis* and *Limiavipes curriei*, as well as prints of Cenozoic avians, with those prints attributed the small theropod trace *Irenichnites* isp. [16]. If size were the sole criterion for distinguishing the tracks of theropods from those of birds, *Limiavipes curriei* and *Wupus agilis*, as well as traces of large Cenozoic avians, if those ichnomorphotypes were to be recovered from the Mesozoic, would erroneously be identified as belonging to a small theropod track-maker. Once the linear data (S1 Table and S2 Table) are log_10_-transformed and the mean values of each variable removed, no print from Limiavipedidae shared the same morphospace as the theropod prints (although one avian print, *Gruipeda maxima*, did occupy the same morphospace as did the prints of small theropods), and no prints from Limiavipedidae were misidentified by the discriminant analysis as belonging to theropods. The seven prints of theropods (one of *Irenesauripus* isp. and six of *Magnoavipes* isp.) were misidentified as belonging to Limiavipedidae were those prints that contain large amounts of missing data.

#### There is no definite delineation in total divarication between the tracks of small theropods and those of large birds

The digits of small theropods are reported to be, in general, less splayed than those of similarly-sized birds (total divarication of 90° or less), but total divarication cannot be used as the sole diagnostic feature, as it is highly variable in Cretaceous small- and medium-sized theropods, and in both Cretaceous and extant avians [28] (Table 3). For example, the "cut-off" value of 100° total divarication for *Magnoavipes denalisensis* is used to attribute these traces to those of a large avian [19]; however, this was based on misinterpretations of work by Lee [17] of *Magnoavipes* isp. traces as avian (rather than theropod) based on comparisons to only average total divarication (rather than examining the range of total divarications) of Cretaceous avian prints [3], [18]. Also, it was determined by Lockley et al. [18] and Matsukawa et al. [28] that many of the diagnostic features of the type trackway of *Magnoavipes lowei* are preservational artifacts due to both the substrate consistency and extramorphologic features [18], [28]. Based on long pace and stride compared to footprint length, high pace angulation, and low footprint rotation, the likely track-makers for *Magnoavipes* isp. are Ornithomimidae (Ornithomimipodidae) [18], [28]. Assigning *Magnoavipes* isp. to Ornithomimidae (Ornithomimipodidae) is supported by the analyses herein. Trackways of *Magnoavipes* isp. display the relatively low FL/PL ratio (0.18) of other trackways of theropods, and all specimens (save those with large amounts of missing data) fall within the “theropod” morphospace (Fig 7). Also, both *Magnoavipes* isp. and the “theropod” morphospace group are significantly different from avian ichnotaxa and the avian morphospace group (Fig 7, [31]). It is only when looking at the maxima of total divarications (Table 3) do we see that traces of large avians have a consistently higher total divarication than do traces of small- and medium-sized theropods, as these values compare favorably with Wright’s [15] observation that the total divarication of theropod footprints is below 90°.

#### Footprint splay alone does not differentiate the tracks of large avians from those of small theropods

In this analysis, footprint splay (FL/FW) is also not sufficient to differentiate the traces of large avians from those of small theropods, although it has previously been used by McCrea and Sarjeant (fig 31.13, [12]) to differentiate the large avian trace, *Limiavipes curriei*, from several dinosaurian ichnotaxa. While, in general, theropod traces have a footprint length that exceeds footprint width (FL/FW > 1.00), at the time of the study [12], 1) there was little overlap in both size and morphology between theropod and avian traces preserved at that site, making their identification unambiguous, and 2) data from Cenozoic avian ichnotaxa provides new information on patterns of footprint splay of large avians tracks. Data presented in Table 3 indicates that the mean FL/FW is similar for both small theropod and large avian traces.

#### The difference in the average footprint length to pace length ratio between tracks of large birds and those of small theropods

It is only when the ratio of footprint length to pace length is examined (Table 4) do we see that the trackways attributed to theropod track-makers display a consistently longer pace compared to footprint length than to those trackways attributed to similarly-sized avians. Pace (or step) lengths may be subjected to biomechanical controls. For example, a large wading avian may have a leg length that is relatively similar to that of a similarly-sized small theropod, but biomechanical differences in locomotion [30] may constrain a large wading bird to taking shorter steps than its theropod counterpart. However, shortened steps may have a behavioral component. Avian traces that are associated with feeding traces have relatively shorter pace lengths than the same traces not associated with feeding traces (*Ignotornis mcconnelli* [32]; *Ignotornis gaijiensis* [33]). Feeding traces do not consistently preserve, and much of the shore- and wading bird behavior exhibited by Cretaceous avian traces might be related to feeding activities. *Wupus agilis*, based on an examination of ichnomorphology, total divarication, footprint splay, FL/PL, and in discriminant analyses, is the most similar to *Limiavipes curriei*, and can confidently be assigned to the ichnofamily Limiavipedidae. Both *Wupus agilis* and *Limiavipes curriei* can be attributed to large, wading avian track-makers.

### Avian diversity during the Early Cretaceous

The revised avian referral for *Wupus agilis* indicates the presence of at least one paleo-ecotype of large wading bird with a functionally tridactyl, (inferred) unwebbed, pes in the Early Cretaceous of China. The track-maker for *Wupus agilis* is distinct from the track-maker of *Limiavipes curriei* from western North America in being a larger bird based, in part, on its interpretation of having longer legs (larger pace) and a larger foot (larger footprint length). *Wupus agilis* lacks hallux impressions and thus can be inferred to be distinct from, and have had a different lifestyle, than the wading birds that made similarly-sized traces with extant heron-like halluces from the Early Cretaceous (Albian) Eumeralla Formation at Dinosaur Cove, southern Australia [34]. Thus, we can conclude that there were at least three distinct morphotypes of large wading birds present during the Early Cretaceous for which skeletal material has not yet been recovered. Assuming that these birds were capable of sustained powered flight, they were probably capable of global distribution throughout Gondwana and Laurasia, similar to extant egrets and herons (Family Ardeidae) that are found in Asia, Australia, and North America.

The results of this study, that 1) *Wupus agilis* is most similar in ichnomorphology to *Limiavipes curriei* and can be reassigned to the ichnofamily Limiavipedidae, and 2) multivariate statistical analyses indicate Limiavipedidae are significantly different from theropod ichnotaxa, and share ichnomorphologic traits with Cretaceous, Cenozoic, and extant avian ichnites, demonstrate that 1) large wading birds had a global distribution in the Early Cretaceous, and 2) it is possible to differentiate between the traces of large wading birds and those of small theropods by using multiple lines of evidence. Future studies that collect and report complete datasets on Mesozoic, Cenozoic, and extant avian ichnites, and ichnites attributed to small theropods, rather than reporting data for the type material and average values only, will aid greatly in discerning improved criteria for differentiating between avian and theropod ichnites, and improve the palaeofaunal data these ichnites provide.

## Supporting Information

S1 Table Linear and angular data of footprints of *Wupus agilis* (Early Cretaceous) from the Lotus Tracksite, Chongqing, China. Linear and angular data collected from the *Wupus agilis* tracks at the Lotus Tracksite. Track # corresponds to individual tracks within the one meter X one meter grid system established on the track surface for the purposes of data collection. For example, C11 refers to grid square C11, and T refers to track, and the number refers to the order in which the footprint was documented within grid square C11. FL, footprint length; FLwPad, footprint length including proximal morphological features of the “heel;” FW, footprint width; L/W, footprint length:footprint width ratio; L, left; III, digit III; R, right; TOT, total divarication. See Fig 3 in text for schematic of footprint measurements.
(DOCX)

S2 Table Linear and angular data of trackways of *Wupus agilis* (Early Cretaceous) from the Lotus Tracksite, Chongqing, China. Linear and angular data collected from the *Wupus agilis* tracks at the Lotus Tracksite. Track # corresponds to individual tracks within the one meter X one meter grid system established on the track surface for the purposes of data collection. For example, C11 refers to grid square C11, and T refers to track, and the number refers to the order in which the footprint was documented within grid square C11. PL, pace length; SL, stride length; FL, footprint length; PA, pace angulation; FR, footprint rotation; TW, trackway width.
(DOCX)
