# Supplementary material for: Reanalysis of Wupus agilis (Early Cretaceous) of Chongqing, China as a Large Avian Trace: Differentiating between Large Bird and Small Non-Avian Theropod Tracks
Source: PLoS One. 2015 May 20;10(5):e0124039. doi: 10.1371/journal.pone.0124039 (PMC4439109; doi:10.1371/journal.pone.0124039)
Supplement: S1 Table — Linear and angular data collected from the Wupus agilis tracks at the Lotus Tracksite. Track # corresponds to individual tracks within the one meter X one meter grid system established on the track surface for the purposes of data collection. For example, C11 refers to grid square C11, and T refers to track, and the number refers to the order in which the footprint was documented within grid square C11. FL, footprint length; FLwPad, footprint length including proximal morphological features of the “heel;” FW, footprint width; L/W, footprint length:footprint width ratio; L, left; III, digit III; R, right; TOT, total divarication. See Fig 3 in text for schematic of footprint measurements. (DOCX) [file pone.0124039.s001.docx]

**S1 Table**. Linear and angular data collected from the *Wupus agilis* tracks at the Lotus Tracksite. Track # corresponds to individual tracks within the meter X meter grid system that was established on the track surface. C11 refers to grid square C11, and T refers to track. FL, footprint length; FLwPad, footprint length including proximal morphological features of the “heel;” FW, footprint width; L/W, footprint length:footprint width ratio; L, left; III, digit III; R, right; TOT, total divarication. See Figure 3 for schematic of footprint measurements.

| **Track #** | **FL (mm)** | **FLwPad (mm)** | **FW (mm)** | **L/W** | **Digit lengths (mm)** | | | **Digit widths (mm)** | | | **Divarication (degrees)** | | |
| --- | --- | --- | --- | --- | --- | --- | --- | --- | --- | --- | --- | --- | --- |
|  |  |  |  |  | **L** | **III** | **R** | **L** | **III** | **R** | **L-III** | **III-R** | **TOT** |
| aa07-T01 | 91 | 91 | 97 | 0.93814 | 75 | 91 | 64 | ? | 14 | 14 | 45 | 40 | 85 |
| aa08-T01 | 90 | 90 | 105 | 0.85714 | 77 | 90 | 62 | 13 | 17 | 18 | 48 | 32 | 80 |
| aa08-T02 | 120 | 120 | 117 | 1.02564 | 85 | 120 | 83 | ? | 17 | 17 | 47 | 47 | 94 |
| aa10-T01 | 100 | 100 | 113 | 0.88496 | 71 | 100 | 65 | 10 | 15 | 15 | 51 | 70 | 121 |
| aa10-T02 | 80 | 80 | ? | ? | 59 | 80 | ? | ? | 11 | ? | 60 | ? | ? |
| A06-T01 | 99 | 99 | 117 | 0.84615 | 80 | 99 | 67 | 10 | 10 | 14 | 53 | 54 | 107 |
| A06-T02 | 116 | 116 | 150 | 0.77333 | 101 | 116 | 78 | 10 | 13 | 13 | 51 | 60 | 111 |
| A06-T03 | 81 | 81 | 100 | 0.81 | 63 | 81 | 65 | 9 | 9 | 7 | 62 | 50 | 112 |
| A06-T04 | 110 | 110 | 130 | 0.84615 | 90 | 110 | 80 | 15 | 21 | 20 | 50 | 50 | 100 |
| A06-T05 | 100 | 100 | 131 | 0.76336 | 85 | 100 | 70 | 15 | 15 | ? | 54 | 63 | 117 |
| A07-T01 | 108 | 108 | 92 | 1.17391 | 81 | 108 | 75 | ? | 9 | 15 | 32 | 38 | 70 |
| A07-T02 | 107 | 107 | 124 | 0.8629 | 96 | 107 | 80 | 20 | 25 | 17 | 44 | 40 | 84 |
| A07-T03 | 110 | 110 | 124 | 0.8871 | 80 | 110 | 74 | ? | 14 | 12 | 43 | 55 | 98 |
| A07-T04 | 90 | 90 | 122 | 0.7377 | 81 | 90 | 78 | 12 | ? | 18 | 46 | 51 | 97 |
| A07-T05 | ? | ? | ? | ? | ? | ? | ? | ? | ? | ? | ? | ? | ? |
| A07-T06 | 92 | 92 | 128 | 0.71875 | 85 | 92 | 90 | ? | 15 | 10 | 48 | 46 | 94 |
| A07-T07 | 94 | 94 | 113 | 0.83186 | 73 | 94 | 61 | ? | 15 | ? | 58 | 53 | 111 |
| A07-T08 | 90 | 90 | 110 | 0.81818 | 81 | 90 | 60 | 8 | 11 | 12 | 51 | 31 | 82 |
| A07-T09 | 82 | 82 | 95 | 0.86316 | 61 | 82 | 60 | 9 | 15 | 14 | 45 | 59 | 104 |
| A08-T01 | ? | ? | 100 | ? | 80 | ? | 62 | 12 | 10 | 15 | 54 | 44 | 98 |
| A08-T02 | 97 | 97 | 113 | 0.85841 | 70 | 97 | 70 | 17 | 15 | 16 | 46 | 48 | 94 |
| A08-T03 | 100 | 100 | 125 | 0.8 | 85 | 100 | 80 | 15 | 20 | 10 | 53 | 31 | 84 |
| A08-T04 | ? | ? | ? | ? | ? | ? | ? | ? | ? | ? | ? | ? | ? |
| A08-T05 | 100 | 100 | 114 | 0.87719 | 80 | 100 | 70 | ? | 15 | ? | 43 | 39 | 82 |
| A08-T06 | 115 | 115 | 123 | 0.93496 | 97 | 115 | 73 | 20 | 20 | 15 | 50 | 45 | 95 |
| A08-T07 | 105 | 105 | 115 | 0.91304 | 80 | 105 | 80 | 13 | 15 | 16 | 48 | 47 | 95 |
| A08-T08 | 94 | 94 | 106 | 0.88679 | 73 | 94 | 75 | 10 | 15 | 17 | 55 | 37 | 92 |
| A08-T09 | 95 | 95 | 112 | 0.84821 | 78 | 95 | 70 | ? | 16 | 15 | 47 | 34 | 81 |
| A08-T10 | 112 | 112 | 145 | 0.772413 | 80 | 112 | 90 | 19 | 15 | ? | 53 | 59 | 112 |
| A08-T11 | ? | ? | ? | ? | ? | ? | ? | ? | ? | ? | ? | ? | ? |
| A09-T01 | 108 | 108 | 135 | 0.8 | 85 | 108 | 90 | ? | 16 | ? | 56 | 59 | 115 |
| A09-T02 | 90 | 90 | 130 | 0.692308 | ? | 90 | ? | ? | ? | ? | ? | ? | ? |
| A09-T03 | 85 | 85 | 105 | 0.809524 | 67 | 85 | 75 | 14 | 17 | 18 | 45 | 55 | 100 |
| A09-T04 | 110 | 110 | 115 | 0.956522 | 85 | 110 | 66 | 20 | 18 | 23 | 46 | 55 | 101 |
| A10-T01 | 137 | 137 | 110 | 1.245455 | 110 | 137 | 107 | 12 | 15 | ? | 35 | ? | ? |
| A10-T02 | 116 | 116 | ? | ? | ? | 116 | 83 | ? | 10 | 15 | ? | 49 | ? |
| A10-T03 | 101 | 101 | 132 | 0.765152 | 79 | 101 | 80 | 13 | 20 | 17 | 63 | 59 | 122 |
| A11-T01 | 94 | 94 | 116 | 0.810345 | 72 | 94 | 68 | 15 | 16 | 10 | 60 | 55 | 115 |
| B05-T01 | 89 | 89 | 113 | 0.787611 | 74 | 89 | 62 | 11 | 14 | 19 | 45 | 62 | 107 |
| B06-T01 | 100 | 100 | 100 | 1 | 64 | 100 | 70 | 10 | 15 | 12 | 42 | 48 | 90 |
| B06-T02 | 87 | 87 | 105 | 0.828571 | 70 | 87 | 70 | 10 | 15 | 11 | 48 | 45 | 93 |
| B07-T01 | 95 | 95 | 87 | 1.091954 | 75 | 95 | 55 | 13 | 14 | 14 | 42 | 33 | 75 |
| B07-T02 | 94 | 94 | 100 | 0.94 | 65 | 94 | 65 | 17 | 15 | 19 | 49 | 46 | 95 |
| B07-T03 | 95 | 95 | 97 | 0.979381 | 77 | 95 | 74 | 22 | 15 | 11 | 35 | 40 | 75 |
| B07-T04 | 93 | 93 | 90 | 1.033333 | 62 | 93 | 62 | 14 | 17 | 11 | 42 | 45 | 87 |
| B07-T05 | ? | ? | ? | ? | ? | ? | ? | ? | ? | ? | ? | ? | ? |
| B07-T06 | 77 | 77 | ? | ? | ? | 77 | 62 | ? | 14 | 15 | ? | 64 | ? |
| B07-T07 | 115 | 115 | 134 | 0.858209 | 88 | 115 | 95 | ? | 16 | 15 | 51 | 46 | 97 |
| B07-T08 | 112 | 112 | 108 | 1.037037 | 88 | 112 | 82 | 13 | 16 | 19 | 44 | 34 | 78 |
| B07-T09 | 118 | 118 | 150 | 0.78667 | 100 | 118 | 84 | 16 | 16 | 11 | 45 | 60 | 105 |
| B07-T10 | ? | ? | ? | ? | ? | ? | ? | ? | ? | ? | ? | ? | ? |
| B07-T11 | 102 | 102 | 120 | 0.85 | 75 | 102 | 71 | 15 | 14 | 16 | 52 | 53 | 105 |
| B07-T12 | 102 | 102 | 117 | 0.87179 | 73 | 102 | 73 | ? | ? | 12 | 51 | 55 | 106 |
| B07-T13i | 105 | 105 | ? | ? | ? | 105 | ? | ? | 19 | 15 | ? | 45 | ? |
| B07-T14 | 72 | 72 | 98 | 0.73469 | 70 | 72 | 59 | 1 | 16 | 17 | 48 | 47 | 95 |
| B07-T15 | 110 | 110 | 115 | 0.95652 | 85 | 110 | 64 | 14 | 16 | 13 | 41 | 49 | 90 |
| B07-T16 | 87 | 87 | 130 | 0.66923 | 77 | 87 | 65 | ? | 10 | 11 | 55 | 62 | 117 |
| B07-T17 | 90 | 90 | 100 | 0.9 | 84 | 90 | 74 | 9 | 16 | 12 | 36 | 43 | 79 |
| B08-T01 | 85 | 85 | 103 | 0.82524 | 80 | 85 | 70 | 17 | 15 | 20 | 44 | 46 | 90 |
| B08-T02 | 95 | 95 | 90 | 1.05556 | 80 | 95 | 83 | ? | ? | ? | 39 | 31 | 70 |
| B08-T03 | 103 | 103 | 134 | 0.76866 | 85 | 103 | 75 | 25 | 20 | 22 | 52 | 68 | 120 |
| B08-T04 | 100 | 100 | 114 | 0.87719 | 77 | 100 | 73 | 14 | 25 | 15 | 51 | 49 | 100 |
| B08-T05 | 100 | 115 | 105 | 0.95238 | 68 | 100 | 72 | 10 | 10 | 12 | 40 | 45 | 85 |
| B08-T06 | ? | ? | ? | ? | ? | ? | 55 | 15 | 20 | 14 | 45 | 40 | 85 |
| B08-T07 | ? | ? | ? | ? | ? | ? | ? | ? | ? | ? | ? | ? | ? |
| B09-T01 | 100 | 100 | 112 | 0.89286 | 87 | 100 | 78 | 19 | 10 | 16 | 45 | 48 | 93 |
| B09-T02 | ? | ? | ? | ? | ? | ? | ? | ? | ? | ? | ? | ? | ? |
| B09-T03 | 106 | 106 | 118 | 0.89831 | 82 | 106 | 83 | 12 | 10 | 13 | 41 | 41 | 82 |
| B09-T04 | 92 | 92 | 108 | 0.85185 | 75 | 92 | 64 | 10 | 12 | 16 | 47 | 34 | 81 |
| B09-T05 | 109 | 109 | 108 | 1.00926 | 82 | 109 | 69 | 10 | 16 | 12 | 42 | 45 | 87 |
| B09-T06 | 112 | 112 | 113 | 0.99115 | 86 | 112 | 72 | 13 | 15 | ? | 37 | 55 | 92 |
| B09-T07 | 120 | 120 | 132 | 0.90909 | 95 | 120 | 92 | 16 | 18 | ? | 45 | 45 | 90 |
| B09-T08 | 126 | 126 | 143 | 0.88112 | 122 | 126 | 81 | ? | 20 | 12 | 40 | 49 | 89 |
| B09-T09 | ? | ? | 125 | ? | ? | ? | ? | ? | 16 | 14 | 51 | 66 | 117 |
| B09-T10 | ? | ? | ? | ? | ? | ? | ? | ? | ? | ? | ? | ? | ? |
| B09-T11 | 115 | 115 | 132 | 0.871212 | 86 | 115 | 85 | 20 | 17 | ? | 52 | 56 | 108 |
| B09-T12 | 92 | 92 | 115 | 0.8 | 71 | 92 | 69 | 15 | 16 | 15 | 56 | 60 | 116 |
| B10-T01 | 98 | 98 | 115 | 0.852174 | 70 | 98 | 79 | 18 | 17 | 27 | 52 | 65 | 117 |
| B10-T02 | 92 | 92 | 105 | 0.876190 | 71 | 92 | 71 | 12 | 11 | 12 | 48 | 45 | 93 |
| B10-T03 | 112 | 112 | ? | ? | ? | 11.2 | 75 | ? | 10 | 10 | 53 | ? | ? |
| B10-T04 | 88 | 88 | 104 | 0.846154 | 61 | 88 | 58 | 10 | 10 | 9 | 65 | 59 | 124 |
| B10-T05 | 110 | 110 | 145 | 0.758621 | 98 | 110 | 85 | ? | ? | ? | 50 | 67 | 117 |
| B10-T07 | 102 | 102 | 123 | 0.829268 | 85 | 102 | 84 | 14 | 14 | ? | 43 | 55 | 98 |
| B10-T08 | 120 | 120 | 145 | 0.827586207 | 105 | 120 | 84 | 10 | 13 | 10 | 49 | 54 | 103 |
| B11-T01 | 94 | 94 | 102 | 0.921569 | 67 | 94 | 70 | 16 | 14 | 20 | 49 | 49 | 98 |
| C04-T01 | 100 | 100 | ? | ? | ? | 100 | 70 | ? | 10 | 10 | ? | 85 | ? |
| C05-T01 | 115 | 115 | 121 | 0.950413 | 80 | 115 | 71 | 10 | 12 | 22 | 46 | 52 | 98 |
| C05-T02 | 94 | 94 | 107 | 0.878505 | 78 | 94 | 67 | 12 | 10 | 15 | 44 | 46 | 90 |
| C05-T03 | ? | ? | ? | ? | ? | ? | ? | ? | ? | ? | ? | ? | ? |
| C06-T01 | 97 | 97 | 110 | 0.881818 | 81 | 97 | 80 | 12 | 14 | 15 | 38 | 42 | 80 |
| C06-T02 | 108 | 108 | 152 | 0.710526 | 97 | 108 | 82 | 15 | 15 | 10 | 58 | 65 | 123 |
| C06-T03 | 90 | 90 | 90 | 1 | 77 | 90 | 87 | 12 | 10 | 15 | 37 | 30 | 67 |
| C06-T04 | ? | ? | ? | ? | ? | ? | ? | ? | ? | ? | ? | 61 | ? |
| C06-T05 | 92 | 92 | 110 | 0.836364 | 75 | 92 | 85 | 14 | 17 | 15 | 42 | 50 | 92 |
| C06-T06m | 100 | 100 | 118 | 0.847458 | 80 | 100 | 77 | 17 | 23 | 17 | 45 | 36 | 81 |
| C07-T01 | 70 | 70 | 93 | 0.752688 | 62 | 70 | 59 | 10 | 9 | 10 | 50 | 53 | 103 |
| C07-T02 | 125 | 125 | 120 | 1.041667 | 105 | 125 | 85 | ? | 15 | 14 | 44 | 41 | 85 |
| C07-T03 | 100 | 100 | ? | ? | ? | 100 | 80 | ? | 15 | 23 | ? | 45 | ? |
| C07-T04 | 113 | 113 | 125 | 0.904 | 92 | 113 | 83 | 18 | 17 | 15 | 45 | 47 | 92 |
| C07-T05 | 84 | 84 | ? | ? | ? | 84 | 65 | ? | 10 | 10 | 37 | ? | ? |
| C07-T06 | 88 | 88 | 97 | 0.907216 | 60 | 88 | 62 | 18 | 13 | 16 | 50 | 52 | 102 |
| C07-T07 | 78 | 78 | 105 | 0.742857 | 65 | 78 | 59 | 15 | ? | 15 | 65 | 50 | 115 |
| C07-T08 | 80 | 80 | 108 | 0.740741 | 75 | 80 | 75 | 15 | 15 | 10 | 47 | 49 | 96 |
| C07-T09 | 105 | 120 | 137 | 0.766423 | 95 | 105 | 75 | 12 | 15 | 20 | 50 | 68 | 118 |
| C07-T10 | 105 | 105 | 114 | 0.921053 | 85 | 105 | 70 | 15 | 15 | ? | 50 | 55 | 105 |
| C07-T11 | 105 | 120 | 137 | 0.766423 | 93 | 105 | 72 | 15 | 17 | 17 | 48 | 65 | 113 |
| C07-T12m | 95 | 107 | 135 | 0.703704 | 80 | 95 | 85 | 20 | 20 | 17 | 50 | 50 | 100 |
| C07-T13 | 120 | 120 | 135 | 0.888889 | 82 | 120 | 87 | 20 | 20 | ? | 50 | 50 | 100 |
| C07-T14 | 103 | 103 | 107 | 0.962617 | 77 | 103 | 70 | 15 | 15 | 17 | 49 | 41 | 90 |
| C07-T15 | 115 | 115 | 115 | 1 | 75 | 115 | 85 | ? | 17 | 20 | 48 | 36 | 84 |
| C08-T02 | ? | ? | ? | ? | ? | ? | ? | ? | ? | ? | ? | ? | ? |
| C08-T03 | 122 | 122 | 140 | 0.871429 | 94 | 122 | 96 | ? | 16 | ? | 45 | 48 | 93 |
| C08-T04 | 124 | 124 | 150 | 0.826667 | 103 | 124 | 106 | 15 | ? | ? | 38 | 49 | 87 |
| C08-T05 | 114 | 114 | 102 | 1.117647 | 80 | 114 | 72 | 12 | 12 | 13 | 40 | 42 | 82 |
| C08-T06 | 120 | 120 | 120 | 1 | 97 | 120 | 86 | 17 | 16 | 21 | 40 | 40 | 80 |
| C08-T07 | 70 | 70 | 118 | 0.593220 | 65 | 70 | 66 | 12 | 13 | 16 | 70 | 47 | 117 |
| C08-T08 | 110 | 110 | 90 | 1.222222 | 65 | 110 | 86 | 11 | 11 | 15 | 35 | 36 | 71 |
| C08-T09 | 109 | 109 | 92 | 1.184783 | 82 | 109 | 72 | 13 | 14 | 14 | 34 | 41 | 75 |
| C08-T10 | 134 | 134 | 130 | 1.030769 | 116 | 134 | 106 | ? | ? | ? | 34 | 41 | 75 |
| C09-T01 | 100 | 100 | 108 | 0.925926 | 81 | 100 | 78 | 10 | 12 | 15 | 45 | 46 | 91 |
| C09-T02 | 80 | 80 | 104 | 0.769231 | 59 | 80 | 64 | 18 | 17 | 20 | 57 | 72 | 129 |
| C09-T03 | 83 | 83 | 118 | 0.703390 | 65 | 83 | 69 | ? | ? | ? | 59 | 73 | 132 |
| C09-T04 | 95 | 95 | 120 | 0.791667 | 63 | 95 | 77 | 17 | 20 | 20 | 57 | 61 | 118 |
| C09-T05 | 78 | 78 | 108 | 0.722222 | 77 | 78 | 73 | 12 | 15 | 19 | 52 | 43 | 95 |
| C09-T06 | 117 | 117 | 155 | 0.754839 | 95 | 117 | 84 | 23 | 10 | 25 | 53 | 71 | 124 |
| C09-T07 | 103 | 103 | 125 | 0.824 | 75 | 103 | 75 | ? | ? | ? | 58 | 65 | 123 |
| C09-T08 | 102 | 102 | 128 | 0.796875 | 92 | 102 | 75 | 15 | 20 | 20 | 52 | 49 | 101 |
| C09-T09 | ? | ? | ? | ? | ? | ? | ? | ? | ? | ? | ? | ? | ? |
| C09-T10 | 118 | 118 | 130 | 0.907692 | 75 | 118 | 85 | ? | ? | ? | 57 | 63 | 120 |
| C10-T01 | 89 | 89 | 120 | 0.741667 | 76 | 89 | 70 | 15 | 10 | 10 | 54 | 69 | 123 |
| C10-T02 | 82 | 82 | 115 | 0.713043 | 78 | 82 | 60 | ? | 12 | 15 | 55 | 67 | 122 |
| C10-T03 | 95 | 95 | 130 | 0.730769 | 81 | 95 | 80 | 10 | 10 | 11 | 55 | 65 | 120 |
| C10-T04 | 96 | 96 | 122 | 0.786885 | 72 | 96 | 72 | 13 | 13 | 15 | 60 | 63 | 123 |
| C11-T01 | 105 | 105 | 120 | 0.875 | 75 | 105 | 85 | 12 | 15 | 14 | 52 | 43 | 95 |
| C11-T02 | 87 | 87 | 94 | 0.925532 | 55 | 87 | 55 | 14 | 10 | 11 | 60 | 60 | 120 |
| D05-T01 | 98 | 98 | 132 | 0.742424 | 90 | 98 | 80 | 15 | 15 | 14 | 59 | 51 | 110 |
| D05-T02 | 95 | 95 | 97 | 0.979381 | 68 | 95 | 68 | 10 | 14 | 10 | 47 | 47 | 94 |
| D06-T01 | 107 | 107 | 125 | 0.856 | 84 | 107 | 100 | ? | 19 | 16 | 44 | 43 | 87 |
| D06-T02 | 96 | 96 | 100 | 0.96 | 86 | 96 | 74 | 11 | 20 | 10 | 49 | 45 | 94 |
| D06-T04 | 83 | 83 | ? | ? | ? | 83 | 67 | ? | 13 | 14 | 51 | ? | ? |
| D06-T05 | 96 | 96 | 102 | 0.941176 | 74 | 96 | 72 | 10 | 12 | 14 | 45 | 40 | 85 |
| D06-T06 | 97 | 97 | 107 | 0.906542 | 82 | 97 | 70 | 20 | 17 | 17 | 37 | 53 | 90 |
| D06-T07 | 116 | 116 | 115 | 1.008696 | 87 | 116 | 79 | 12 | 16 | 16 | 41 | 49 | 90 |
| D06-T08 | 125 | 125 | 122 | 1.024590 | 102 | 125 | 88 | 22 | 11 | 18 | 39 | 38 | 77 |
| D06-T09 | 108 | 108 | 88 | 1.227273 | 60 | 108 | 77 | 20 | 17 | 20 | 27 | 49 | 76 |
| D06-T10 | 103 | 103 | 98 | 1.051020 | 72 | 103 | 76 | 12 | 13 | 18 | 39 | 39 | 78 |
| D07-T01 | 114 | 114 | 127 | 0.897638 | 84 | 114 | 72 | 18 | 15 | 16 | 48 | 58 | 106 |
| D07-T02 | ? | ? | ? | ? | ? | ? | ? | ? | ? | ? | ? | ? | ? |
| D07-T03 | 108 | 108 | 118 | 0.915254 | 90 | 108 | 90 | 21 | 22 | 19 | 51 | 57 | 108 |
| D07-T04 | 122 | 122 | ? | ? | ? | 122 | 90 | ? | 16 | 16 | ? | 42 | ? |
| D07-T05 | ? | ? | ? | ? | ? | ? | ? | ? | ? | ? | ? | ? | ? |
| D07-T06 | ? | ? | ? | ? | ? | ? | ? | ? | ? | ? | ? | 37 | ? |
| D08-T02 | 111 | 111 | 125 | 0.888 | 85 | 111 | 90 | 13 | 15 | 21 | 37 | 42 | 79 |
| D08-T03 | 98 | 108 | 117 | 0.837607 | 90 | 98 | 86 | 33 | 15 | 19 | 42 | 42 | 84 |
| D08-T04 | 95 | 95 | 130 | 0.730769 | 87 | 95 | 80 | 16 | 16 | 15 | 42 | 68 | 110 |
| D08-T05 | 114 | 130 | ? | ? | ? | 114 | 82 | ? | 13 | 29 | ? | 55 | ? |
| D08-T06 | 108 | 108 | 82 | 1.317073 | 67 | 108 | 61 | 10 | 14 | 11 | 43 | 38 | 81 |
| D09-T01 | 115 | 115 | 140 | 0.821429 | 97 | 115 | 97 | 27 | 33 | 19 | 51 | 47 | 98 |
| D09-T02i | 116 | 116 | ? | ? | ? | 116 | 77 | ? | 16 | 14 | ? | 43 | ? |
| D09-T03 | 103 | 103 | 123 | 0.837398 | 90 | 103 | 83 | 15 | 20 | 25 | 41 | 49 | 90 |
| D09-T05 | 125 | 133 | 137 | 0.912409 | 90 | 125 | 84 | 21 | 15 | 23 | 50 | 54 | 104 |
| D10-T01 | 92 | 92 | ? | ? | 75 | 92 | ? | 13 | 17 | ? | 69 | ? | ? |
| D10-T02 | 100 | 100 | 113 | 0.884956 | 69 | 100 | 80 | 15 | 15 | 16 | 52 | 50 | 102 |
| D10-T03 | 118 | 118 | 129 | 0.914729 | 97 | 118 | 90 | 26 | 29 | 26 | 41 | 65 | 106 |
| D10-T04 | 105 | 105 | 119 | 0.882353 | 86 | 105 | 86 | 17 | 20 | 22 | 43 | 65 | 108 |
| D10-T05 | 89 | 110 | 122 | 0.729508 | 75 | 89 | 80 | 23 | 20 | 20 | 48 | 54 | 102 |
| D10-T06 | ? | ? | ? | ? | ? | ? | ? | ? | 17 | 12 | ? | 49 | ? |
| D10-T07 | ? | ? | ? | ? | ? | ? | ? | ? | ? | ? | ? | ? | ? |
| D10-T08 | ? | ? | ? | ? | ? | ? | ? | ? | ? | ? | ? | ? | ? |
| D10-T09 | 111 | 111 | ? | ? | ? | 111 | 80 |  | 13 | 18 | ? | 48 | ? |
| E04-T01 | 111 | 111 | 150 | 0.74 | 82 | 111 | 89 | 27 | 23 | 27 | 75 | 54 | 129 |
| E05-T01 | 98 | 98 | 107 | 0.915888 | 78 | 98 | 72 | 10 | 15 | 20 | 45 | 47 | 92 |
| E05-T02 | 83 | 83 | 97 | 0.855670 | 62 | 83 | 59 | 11 | 10 | 15 | 57 | 48 | 105 |
| E05-T03 | 97 | 97 | 100 | 0.97 | 81 | 97 | 70 | ? | 15 | 15 | 40 | 43 | 83 |
| E05-T04 | ? | ? | 97 | ? | 72 | ? | 62 | 14 | 17 | 12 | 47 | 46 | 93 |
| E06-T01 | 102 | 102 | 117 | 0.871795 | 90 | 102 | 89 | ? | ? | ? | 36 | 41 | 77 |
| E06-T02 | ? | ? | 90 | ? | ? | ? | ? | ? | ? | 10 | 45 | 35 | 80 |
| E06-T03 | 96 | 96 | 93 | 1.032258 | 79 | 96 | 61 | ? | 17 | 12 | 42 | 39 | 81 |
| E07-T01 | 98 | 98 | ? | ? | 82 | 98 | ? | 11 | 16 | ? | 43 | ? | ? |
| E07-T02 | ? | ? | ? | ? | ? | ? | ? | ? | ? | ? | ? | ? | ? |
| E09-T01 | 105 | 105 | 117 | 0.897436 | 92 | 105 | 74 | ? | 17 | 20 | 45 | 47 | 92 |
| E09-T02 | 115 | 115 | 136 | 0.845588 | 93 | 115 | 82 | 24 | 21 | ? | 49 | 51 | 100 |
| E09-T03 | 124 | 124 | 146 | 0.849315 | 96 | 124 | 100 | 24 | 17 | 17 | 50 | 44 | 94 |
| E09-T04 | ? | ? | ? | ? | ? | ? | ? | ? | ? | ? | ? | ? | ? |
| E09-T05 | 122 | 122 | 134 | 0.910448 | 95 | 122 | 87 | 19 | 23 | 17 | 46 | 46 | 92 |
| E10-T01 | 100 | 100 | ? | ? | 88 | 100 | ? | 18 | 15 | ? | 48 | ? | ? |
