# Supplementary material for: Reanalysis of Wupus agilis (Early Cretaceous) of Chongqing, China as a Large Avian Trace: Differentiating between Large Bird and Small Non-Avian Theropod Tracks
Source: PLoS One. 2015 May 20;10(5):e0124039. doi: 10.1371/journal.pone.0124039 (PMC4439109; doi:10.1371/journal.pone.0124039)
Supplement: S2 Table — Linear and angular data collected from the Wupus agilis tracks at the Lotus Tracksite. Track # corresponds to individual tracks within the one meter X one meter grid system established on the track surface for the purposes of data collection. For example, C11 refers to grid square C11, and T refers to track, and the number refers to the order in which the footprint was documented within grid square C11. PL, pace length; SL, stride length; FL, footprint length; PA, pace angulation; FR, footprint rotation; TW, trackway width. (DOCX) [file pone.0124039.s002.docx]

**S2 Table:** *Wupus agilis* trackway data from the Lotus Tracksite. PL, pace length; SL, stride length; FL, footprint length; PA, pace angulation; FR, footprint rotation; TW, trackway width. See Figure 3 in text for a schematic of the linear and angular measurements.

| **Trackway number** | **Grid** | **Footprint #** | **Series in trackway** | **PL (mm)** | **SL (mm)** | **FL (mm)** | **PL (mm)** | **PL/FL** |
| --- | --- | --- | --- | --- | --- | --- | --- | --- |
| I | D10 | T09 | 1 | ? | ? | 111 | 58.5 | 0.527027 |
| I | C10 | T01 | 2 | 58.5 | ? | 89 | 58.5 | 0.657303 |
| I | C10 | T04 | 3 | 54.5 | 113.5 | 96 | 54.5 | 0.567708 |
| I | B10 | T02 | 4 | 31.5 | 86 | 92 | 31.5 | 0.342391 |
| I | B10 | T05 | 5 | 30 | 61 | 110 | 30 | 0.272727 |
| I | B10 | T07 | 6 | 39.5 | 67.5 | 102 | 39.5 | 0.387255 |
| II | E09 | T02 | 1 | ? | ? | 115 | 33.5 | 0.291304 |
| II | D09 | T01 | 2 | 33.5 | ? | 115 | 33.5 | 0.291304 |
| II | D09 | T03 | 3 | 31 | 62.5 | 103 | 31 | 0.300971 |
| II | D09 | T05 | 4 | 38.5 | 65 | 125 | 38.5 | 0.308 |
| II | C09 | T10 | 5 | 35 | 70.5 | 118 | 35 | 0.29661 |
| II | C09 | T05 | 6 | 44.5 | 79 | 78 | 44.5 | 0.570513 |
| II | C09 | T08 | 7 | 34.5 | 76.5 | 102 | 34.5 | 0.338235 |
| III | E08 | ? | 1 | ? | ? | ? | 63 | ? |
| III | D08 | T02 | 2 | 63 | ? | 111 | 63 | 0.567568 |
| III | D08 | T04 | 3 | 48.5 | 110.5 | 95 | 48.5 | 0.510526 |
| III | C08 | T03 | 4 | 47.5 | 96.5 | 122 | 47.5 | 0.389344 |
| III | C08 | T10 | 5 | 50 | 97.5 | 134 | 50 | 0.373134 |
| IV | E06 | T04 | 1 | ? | ? | 115 | 25.5 | 0.221739 |
| IV | E06 | T02 | 2 | 25.5 | ? | ? | 25.5 | ? |
| IV | D06 | T02 | 3 | 23 | 48.5 | 96 | 23 | 0.239583 |
| IV | D06 | T05 | 4 | 31 | 54 | 96 | 31 | 0.322917 |
| IV | D06 | T06 | 5 | 32 | 63 | 97 | 32 | 0.329897 |
| IV | D06 | T07 | 6 | 27 | 57.5 | 116 | 27 | 0.232759 |
| IV | C06 | T01 | 7 | 34 | 60.5 | 97 | 34 | 0.350515 |
| IV | C06 | T? | 8 | ? | ? | ? | ? | ? |
| IV | C06 | T06 | 9 | ? | 62 | 100 | ? | ? |
